# Supplementary material for: Unified divergent strategy towards the total synthesis of the three sub-classes of hasubanan alkaloids
Source: Nat Commun. 2021 Jan 4;12:36. doi: 10.1038/s41467-020-20274-1 (PMC7782686; doi:10.1038/s41467-020-20274-1)
Supplement: Supplementary file 1 — Supplementary Information [file 41467_2020_20274_MOESM1_ESM.pdf]

Supplementary Information

Unified Divergent Strategy towards the Total Synthesis of the Three Sub-classes of Hasubanan Alkaloids

Guang Li, Qian Wang and Jieping Zhu*

Affiliations:

Laboratory of Synthesis and Natural Products, Institute of Chemical Sciences and Engineering, Ecole Polytechnique Fédérale de Lausanne, EPFL-SB-ISIC-LSPN, BCH5304, CH-1015 Lausanne (Switzerland)

E-mail: jieping.zhu@epfl.ch

Supplementary Figures

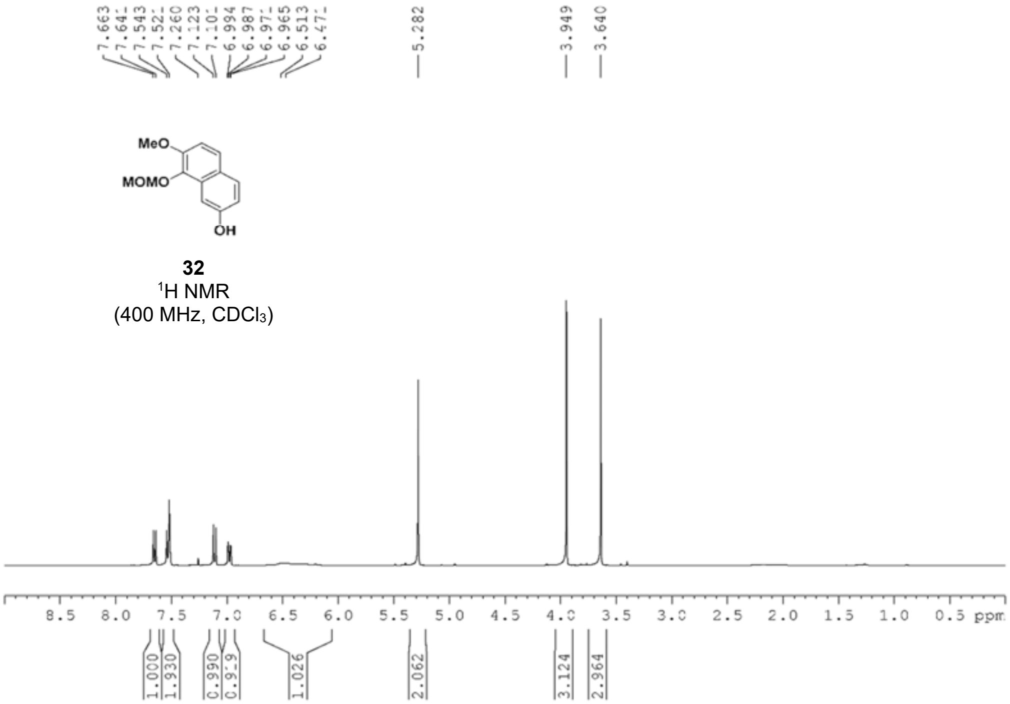

Supplementary Figure 1. ^{1}H NMR spectrum of **32**

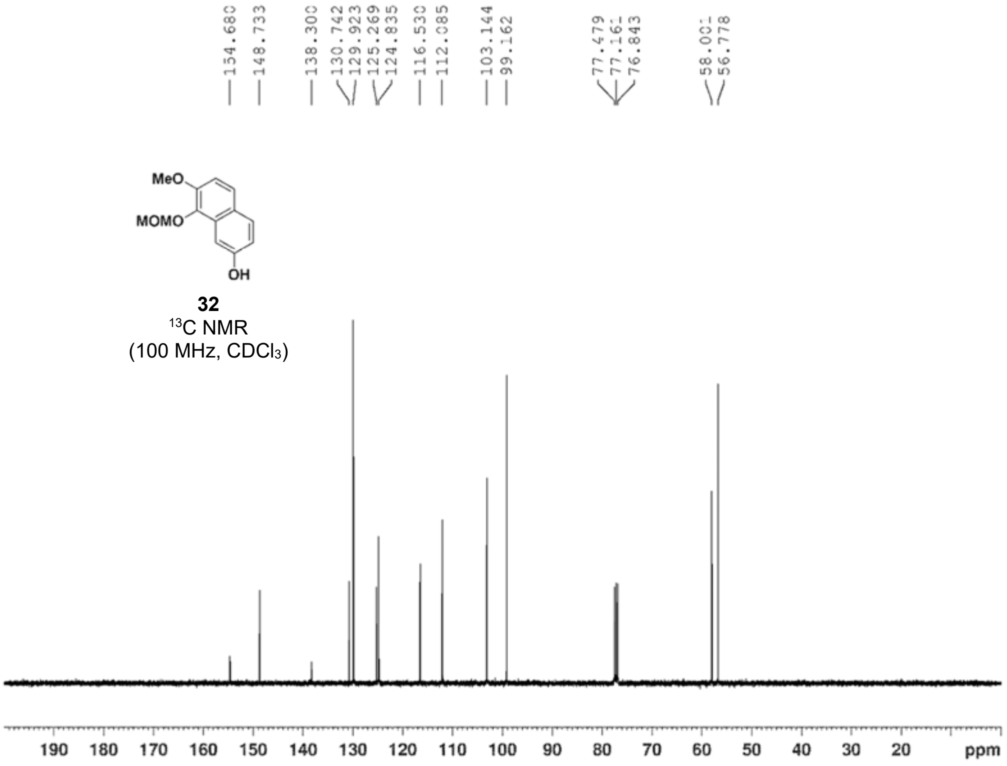

Supplementary Figure 2. ^{13}C NMR spectrum of **32**

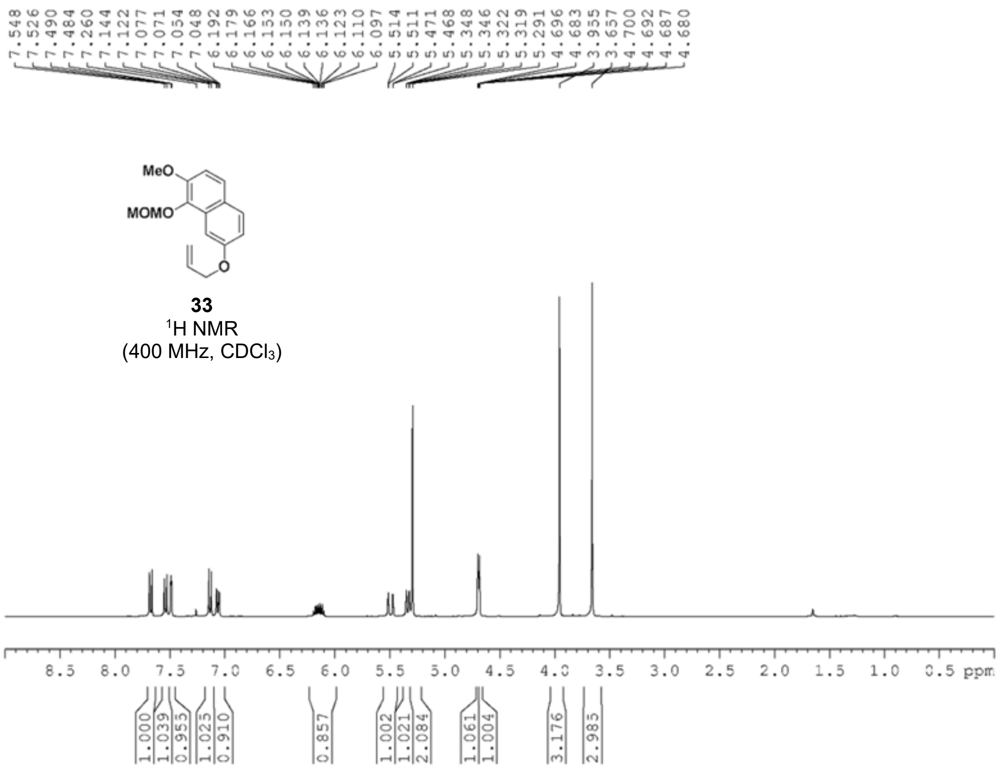

Supplementary Figure 3. ^{1}H NMR spectrum of **33**

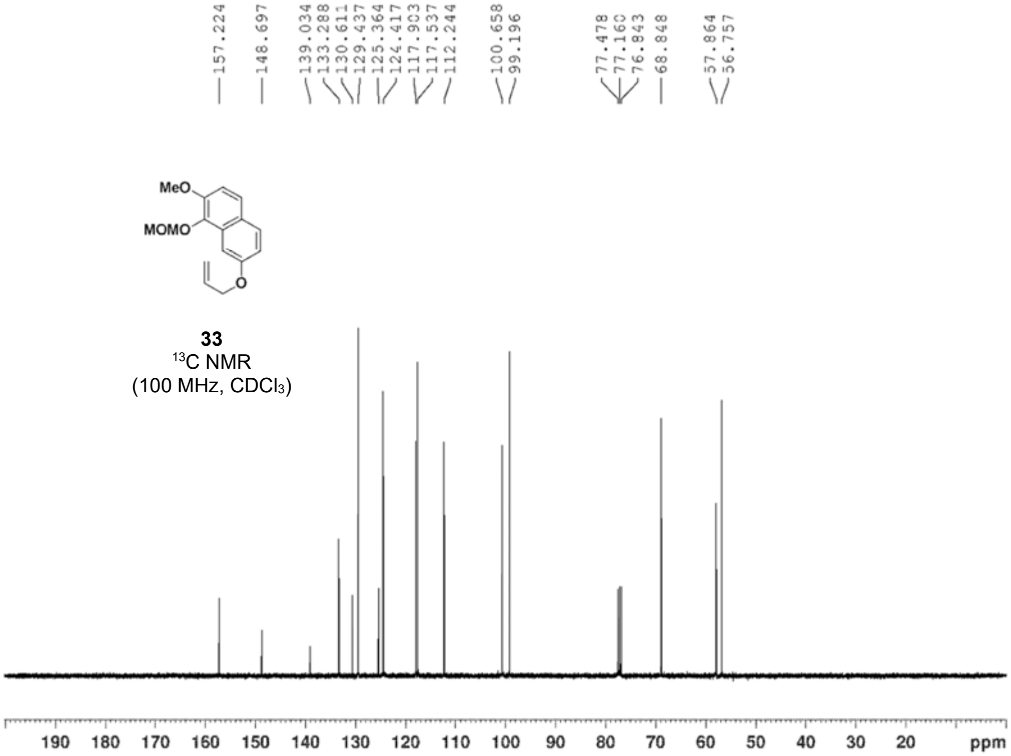

Supplementary Figure 4. ^{13}C NMR spectrum of **33**

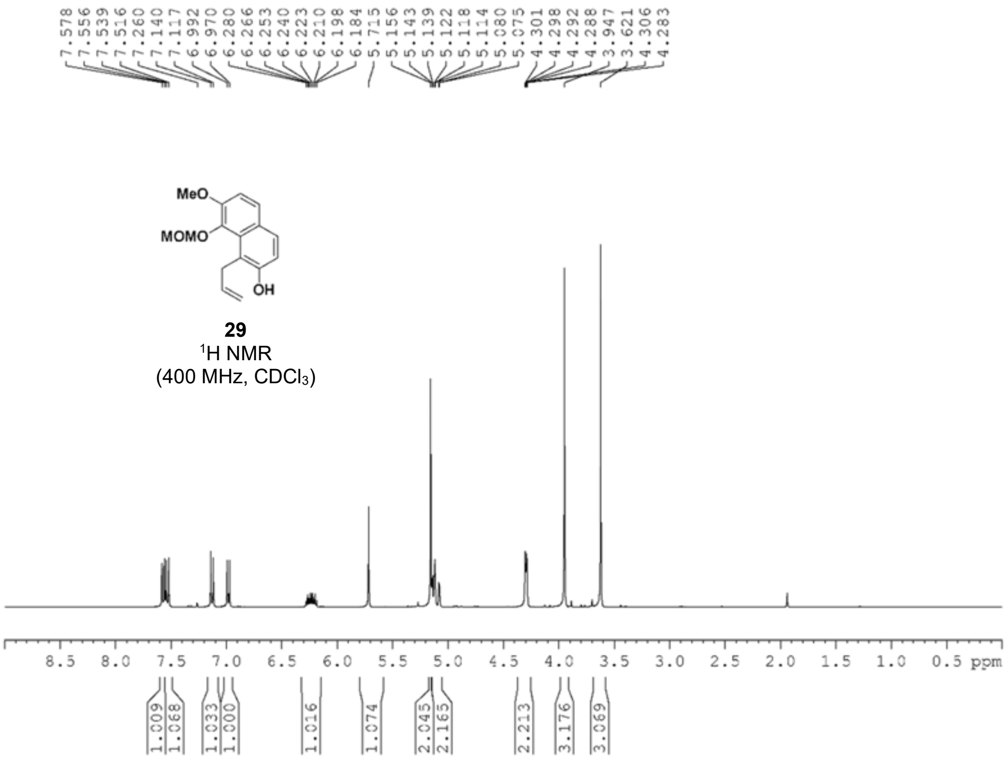

Supplementary Figure 5. ^{1}H NMR spectrum of **29**

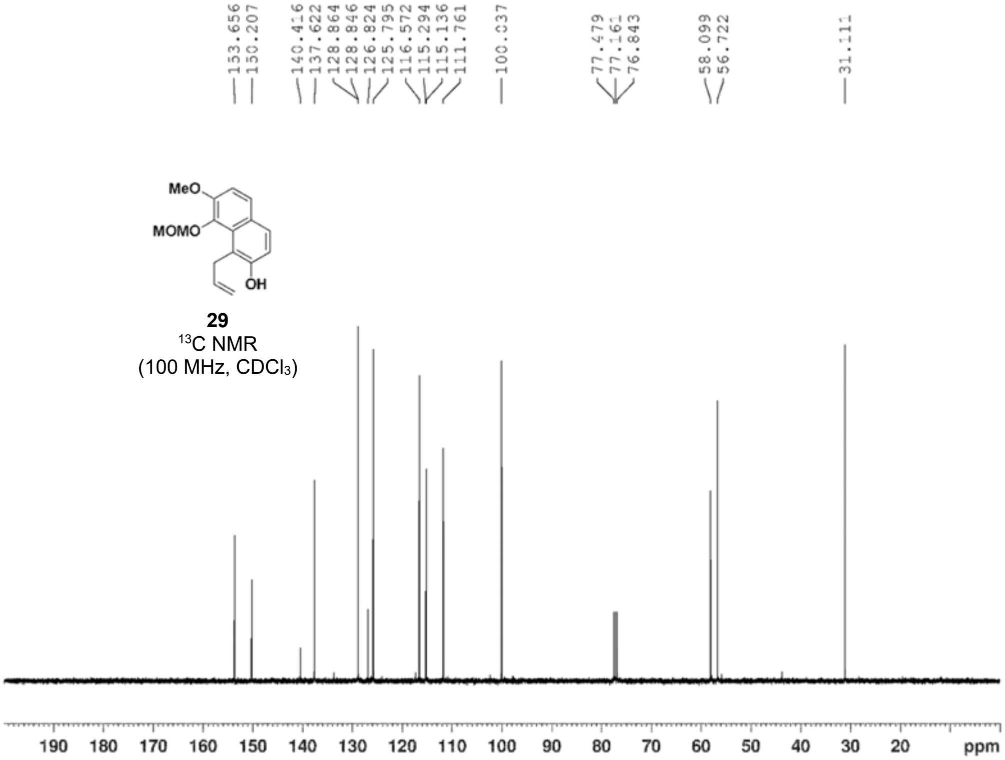

Supplementary Figure 6. ^{13}C NMR spectrum of **29**

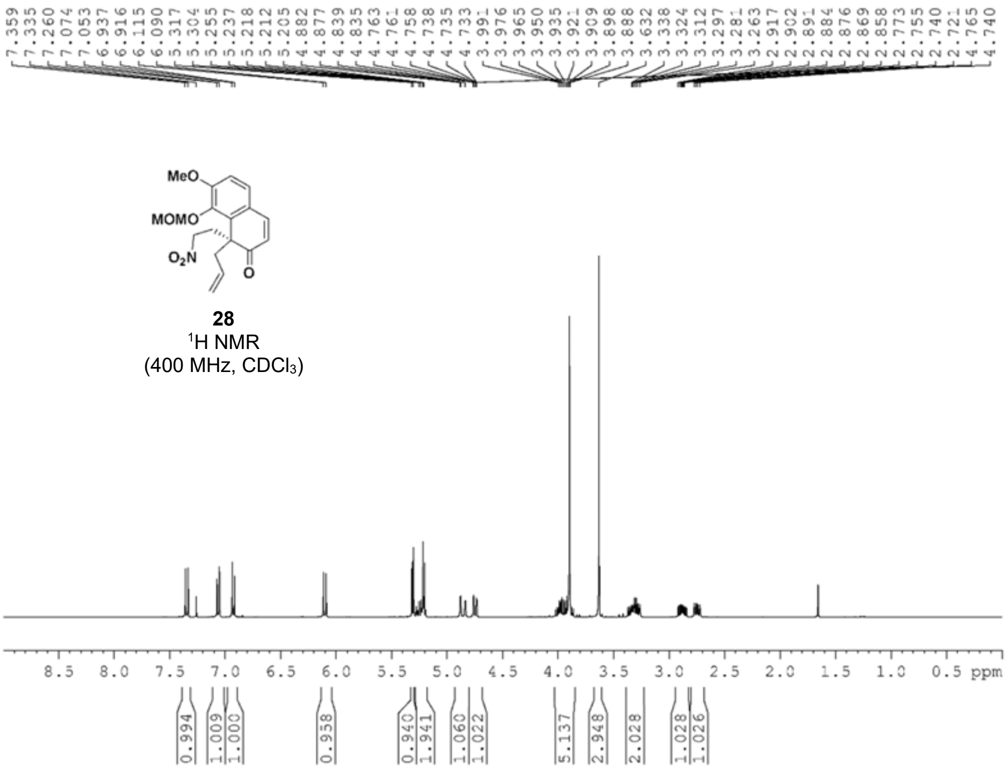

Supplementary Figure 7. ^{1}H NMR spectrum of **28**

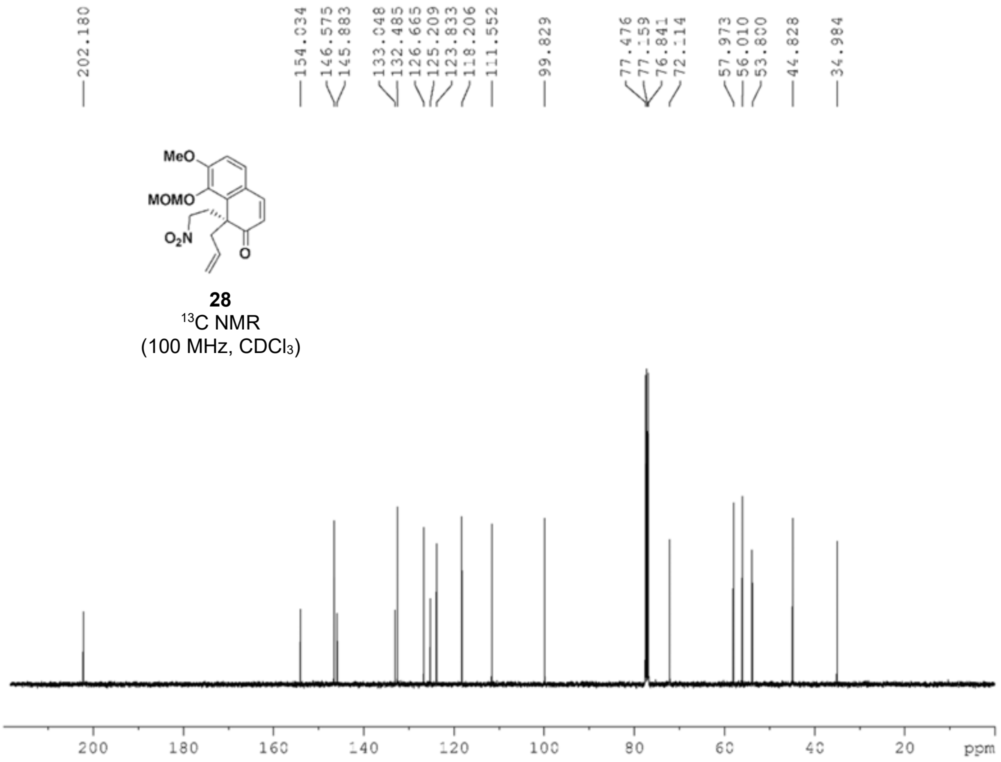

Supplementary Figure 8. ^{13}C NMR spectrum of **28**

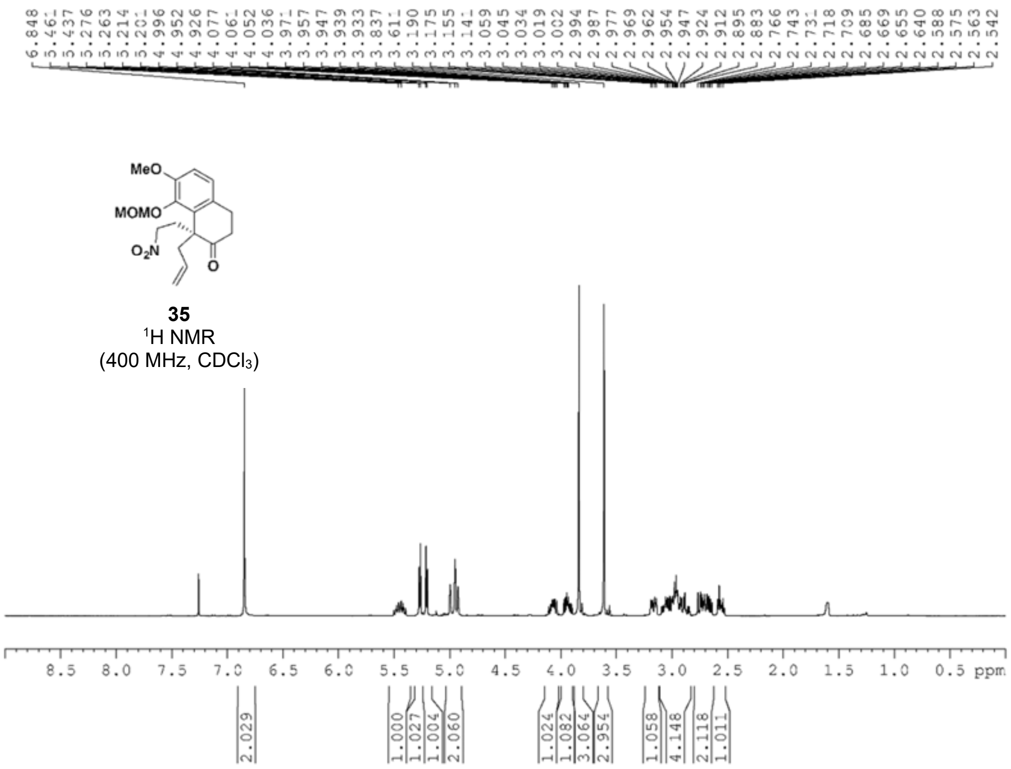

Supplementary Figure 9. ^{1}H NMR spectrum of **35**

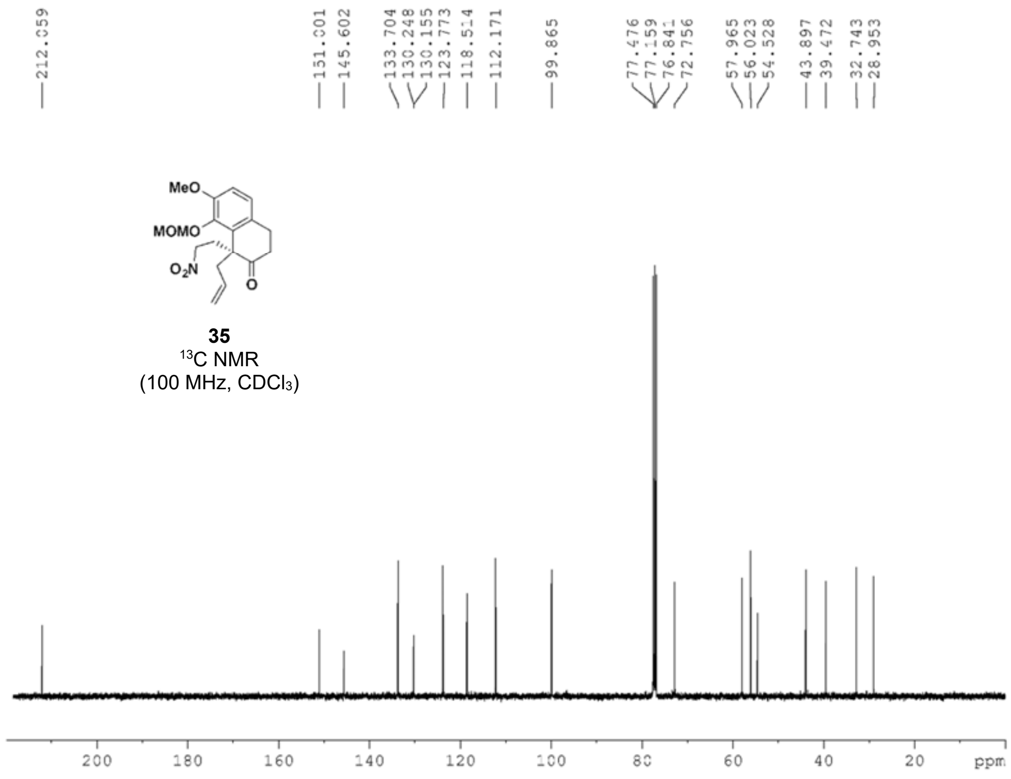

Supplementary Figure 10. ^{13}C NMR spectrum of **35**

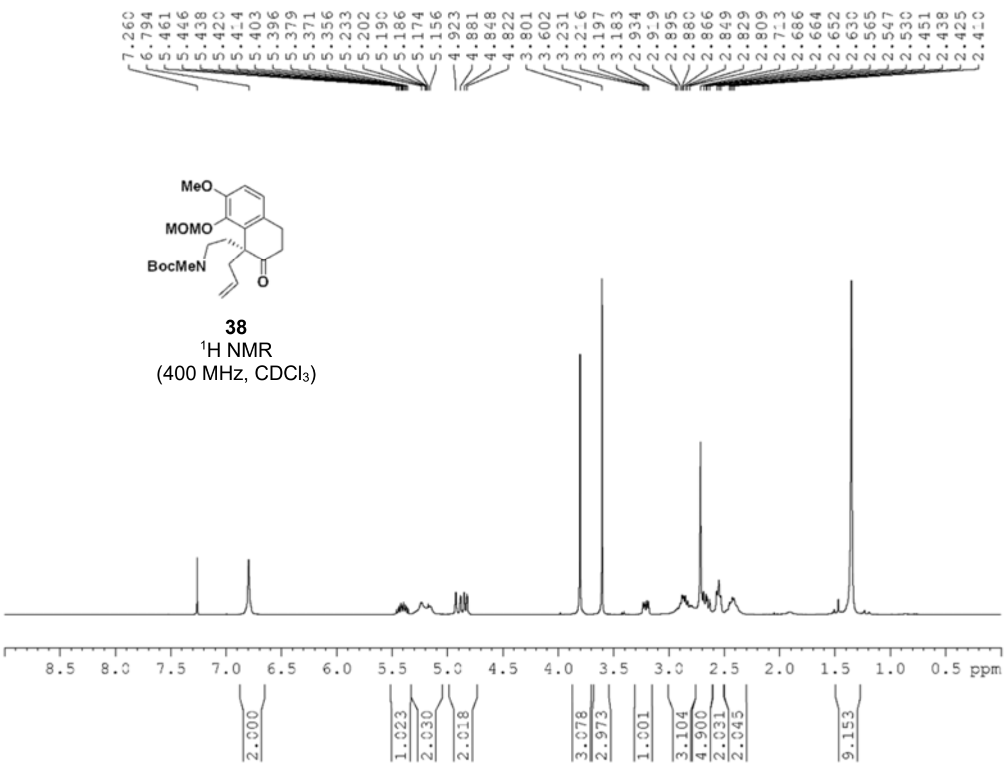

Supplementary Figure 11. ^{1}H NMR spectrum of **38**

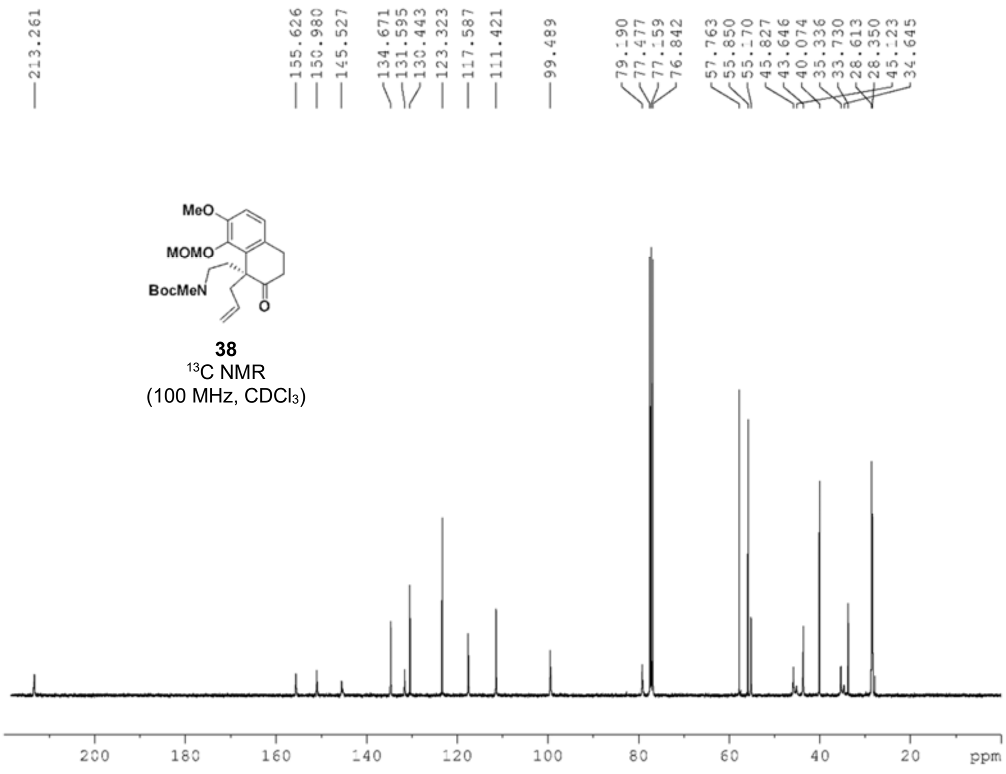

Supplementary Figure 12. ^{13}C NMR spectrum of **38**

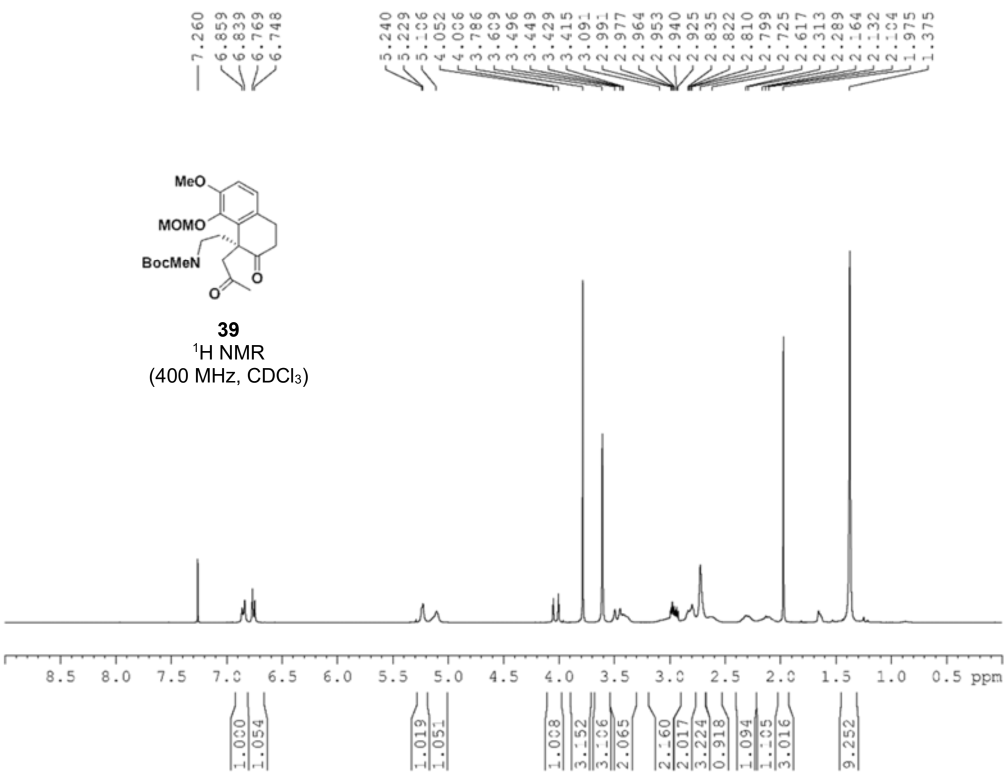

Supplementary Figure 13. ^{1}H NMR spectrum of **39**

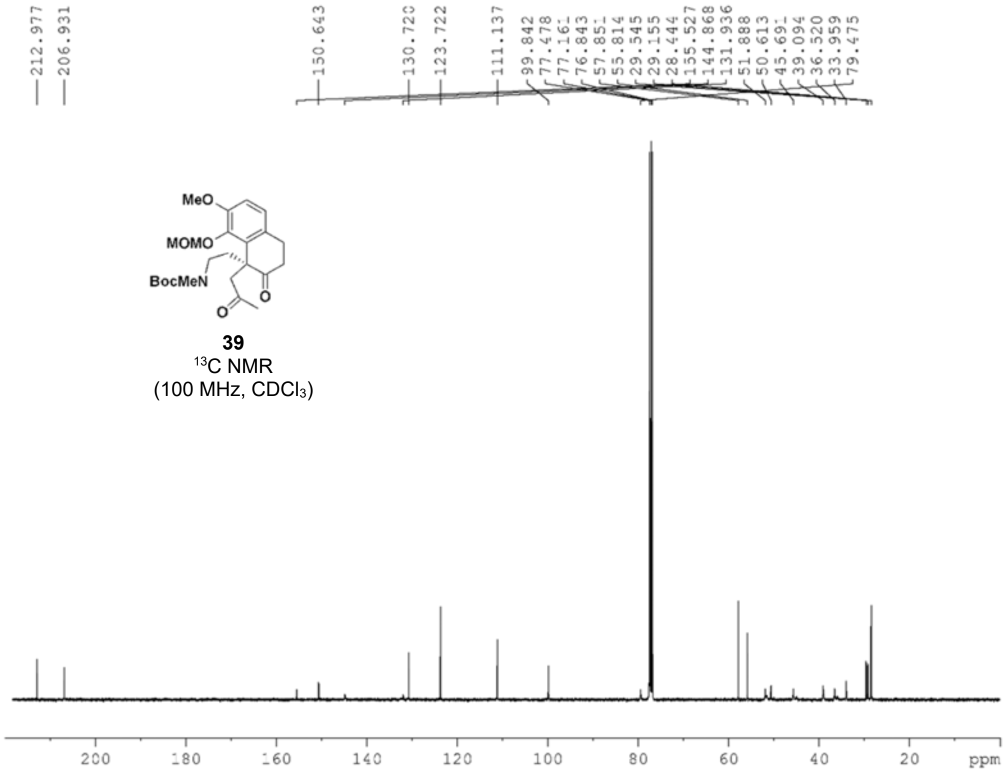

Supplementary Figure 14. ^{13}C NMR spectrum of **39**

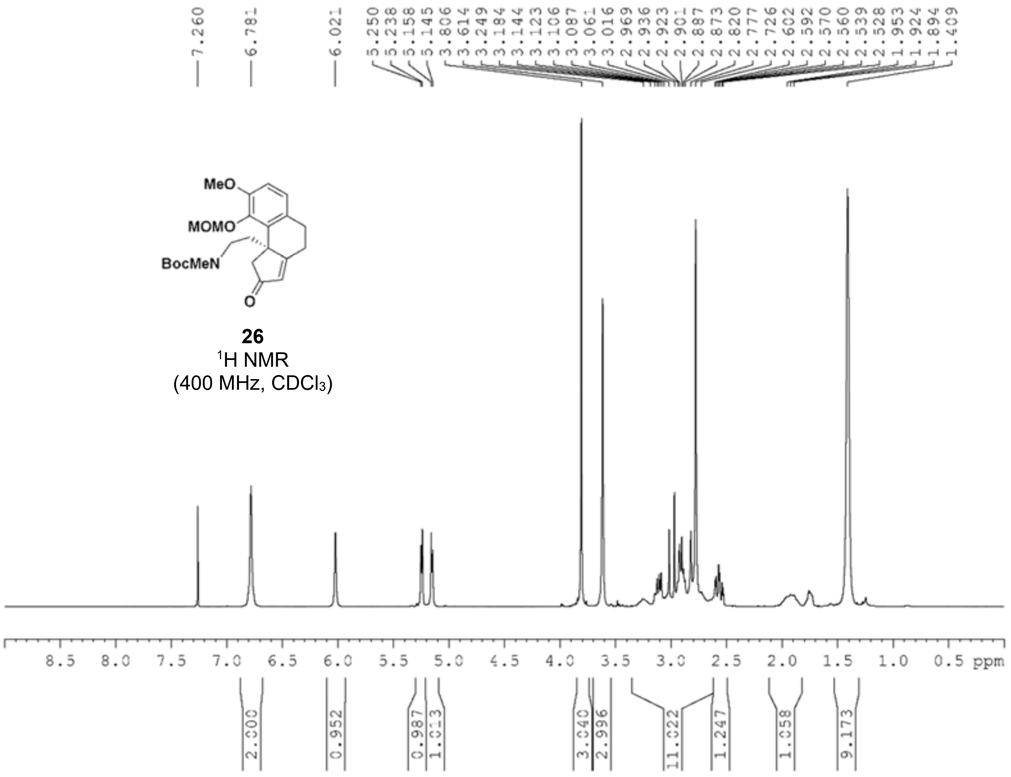

Supplementary Figure 15. ^{1}H NMR spectrum of **26**

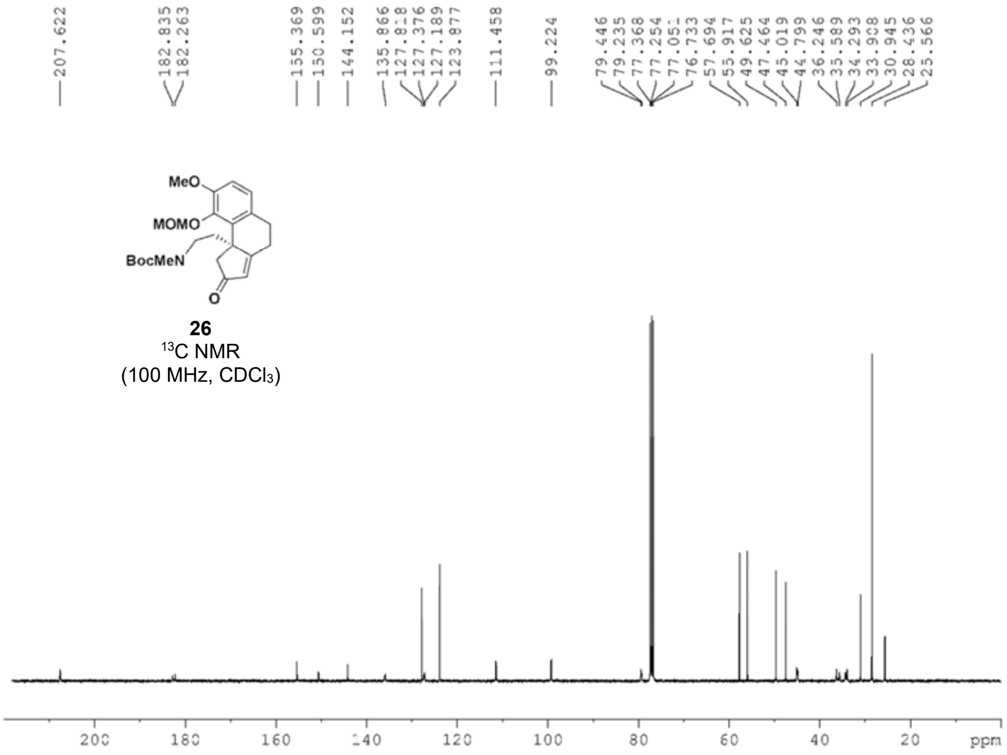

Supplementary Figure 16. ^{13}C NMR spectrum of **26**

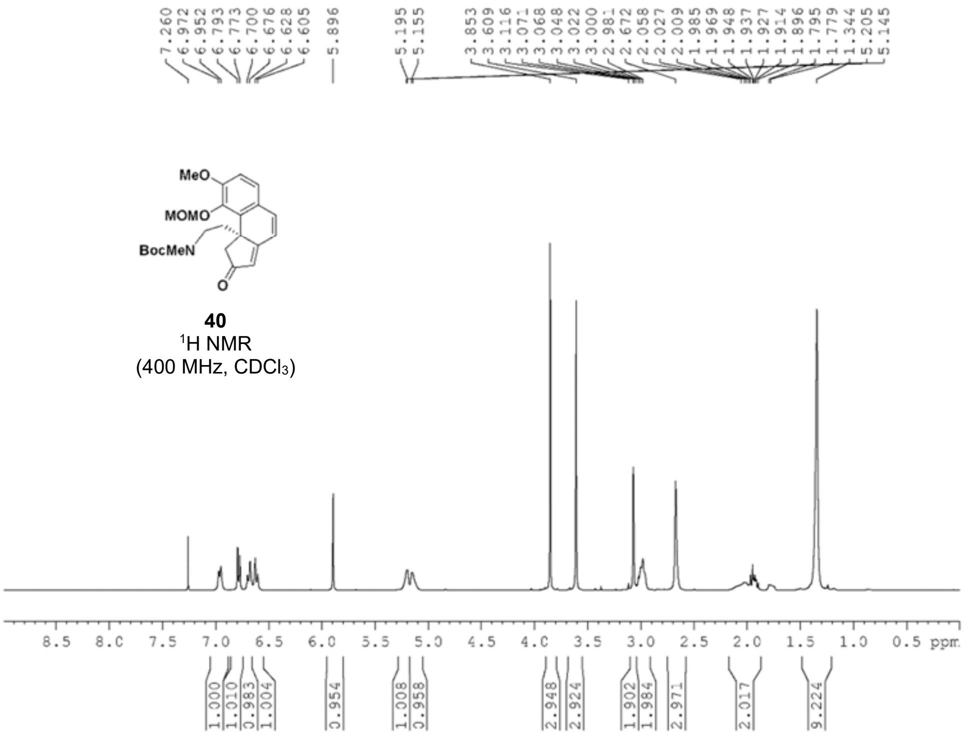

Supplementary Figure 17. ^{1}H NMR spectrum of **40**

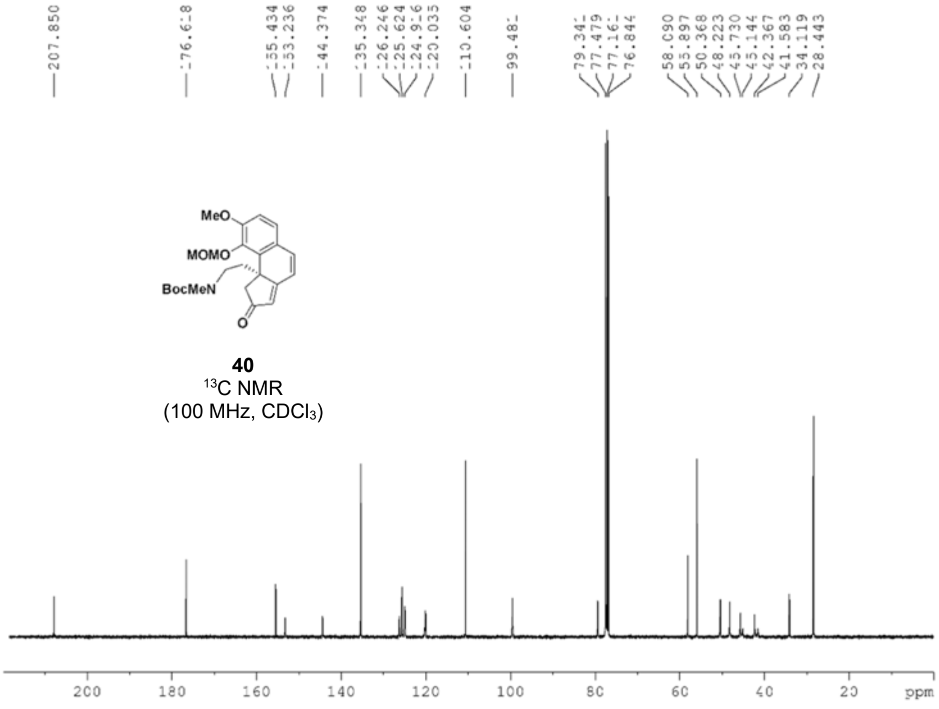

Supplementary Figure 18. ^{13}C NMR spectrum of **40**

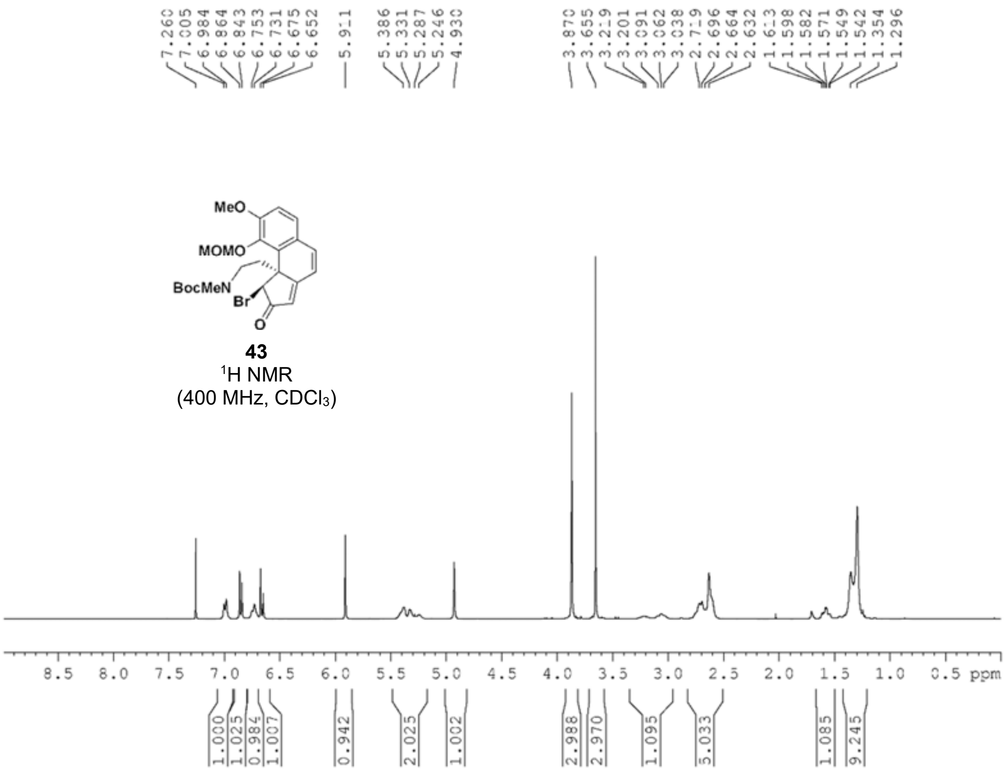

Supplementary Figure 19. ^{1}H NMR spectrum of **43**

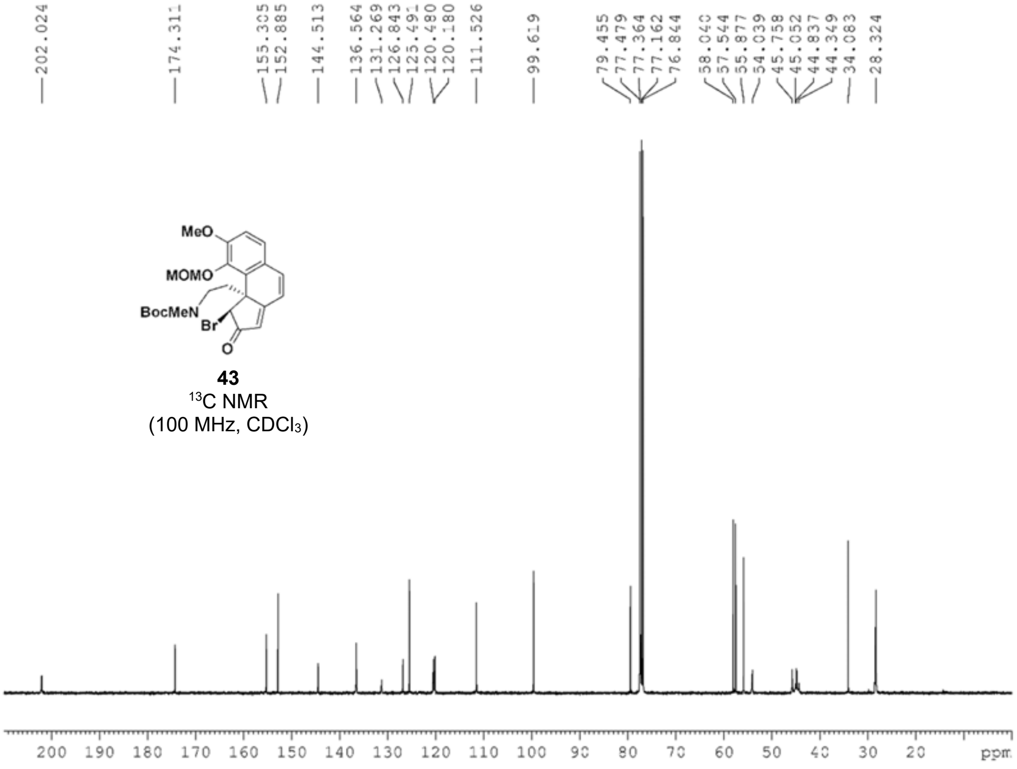

Supplementary Figure 20. ^{13}C NMR spectrum of **43**

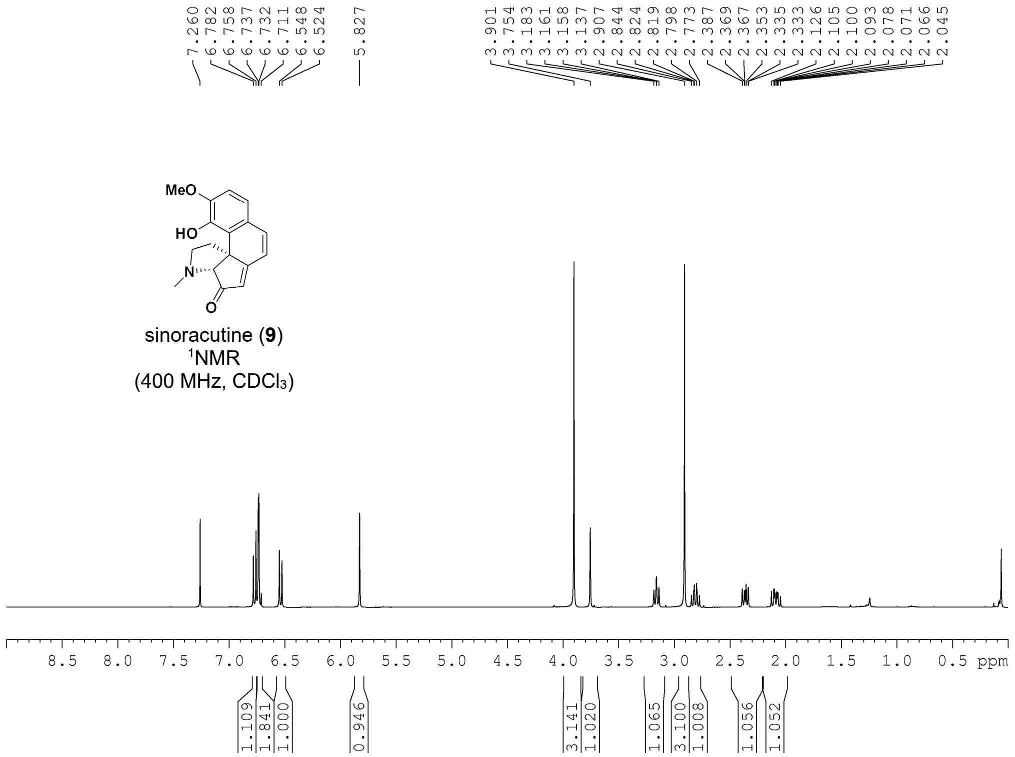

Supplementary Figure 21. ^{1}H NMR spectrum of sinoracutine (**9**)

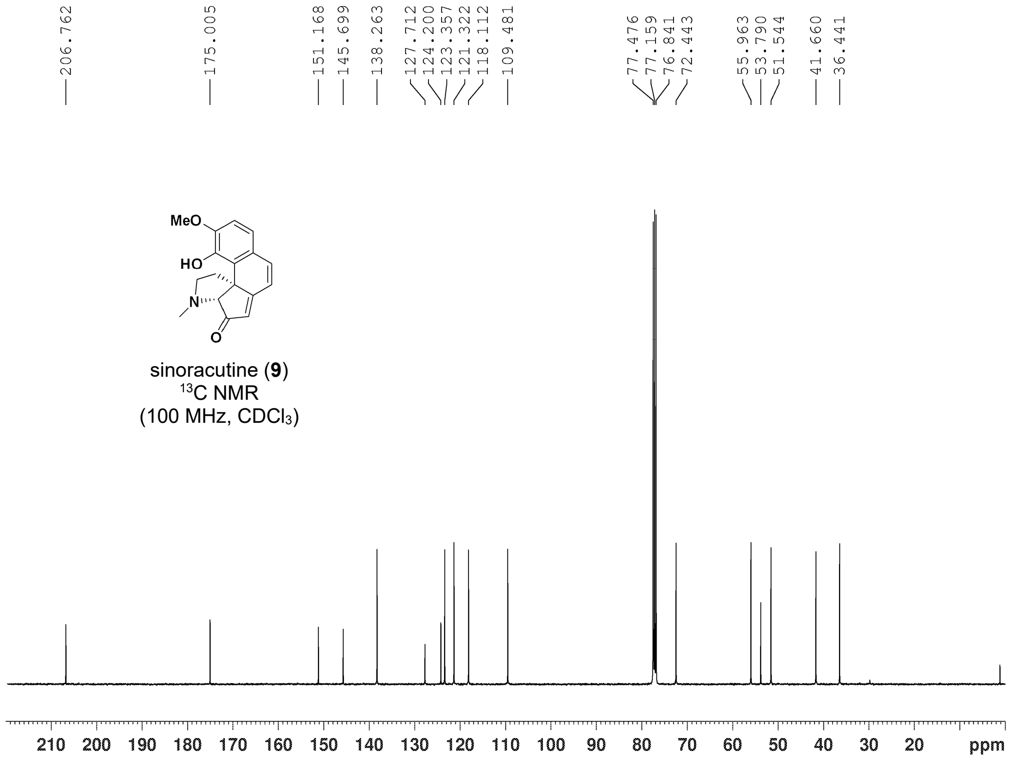

Supplementary Figure 22. ^{13}C NMR spectrum of sinoracutine (**9**)

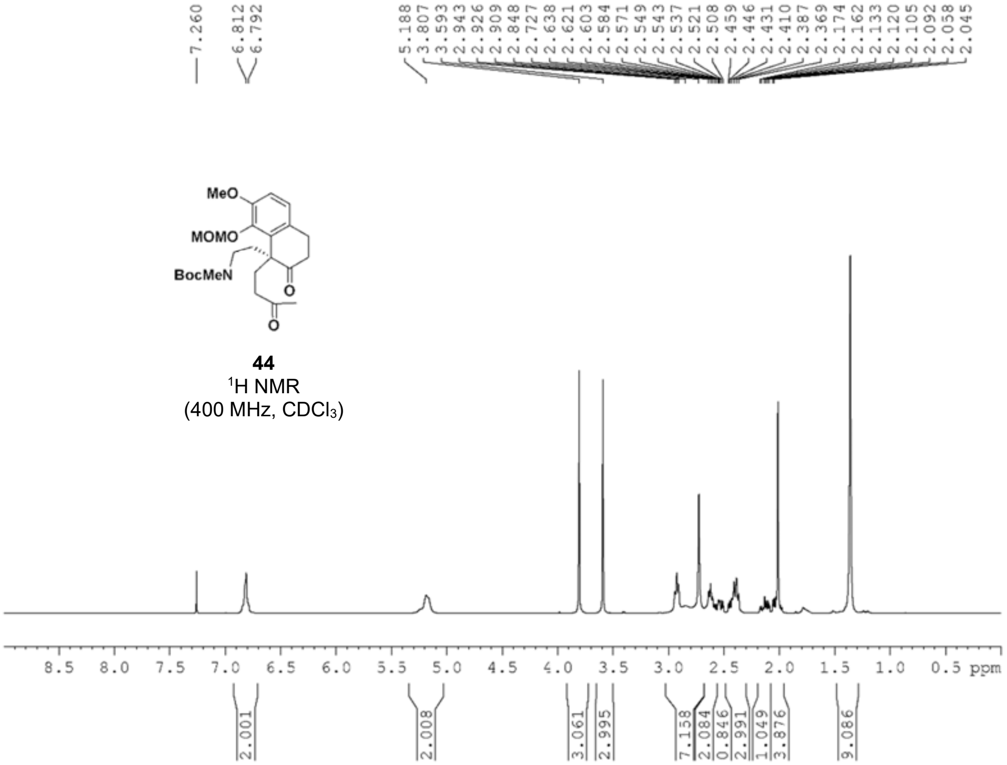

Supplementary Figure 23. ^{1}H NMR spectrum of **44**

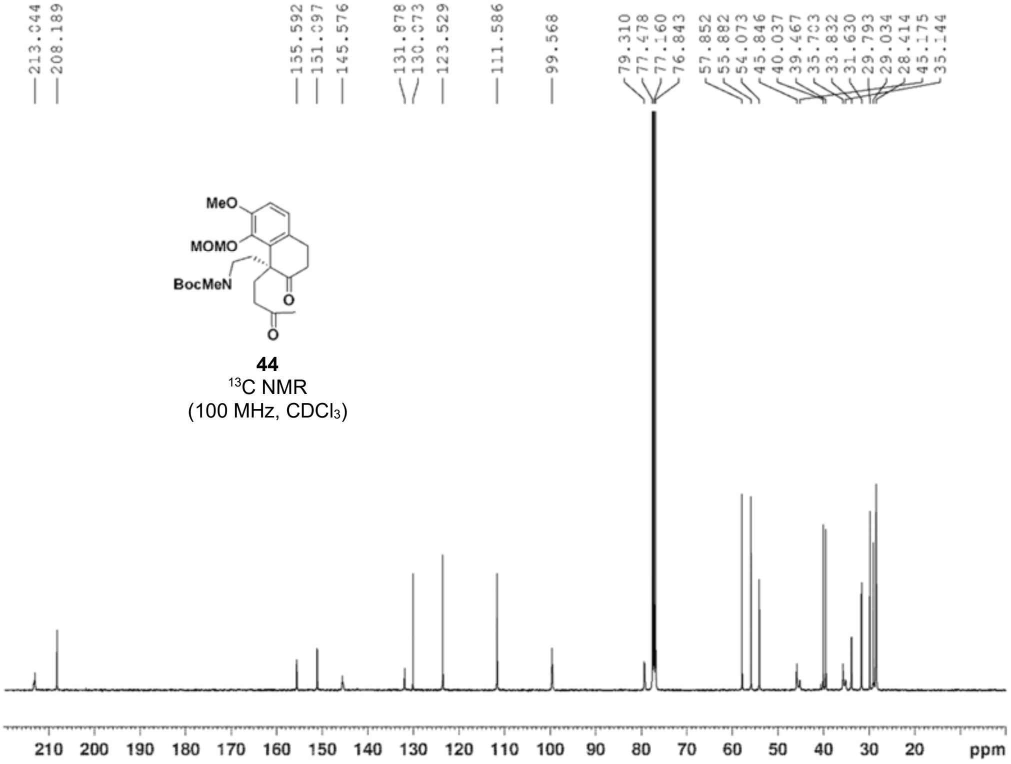

Supplementary Figure 24. ^{13}C NMR spectrum of **44**

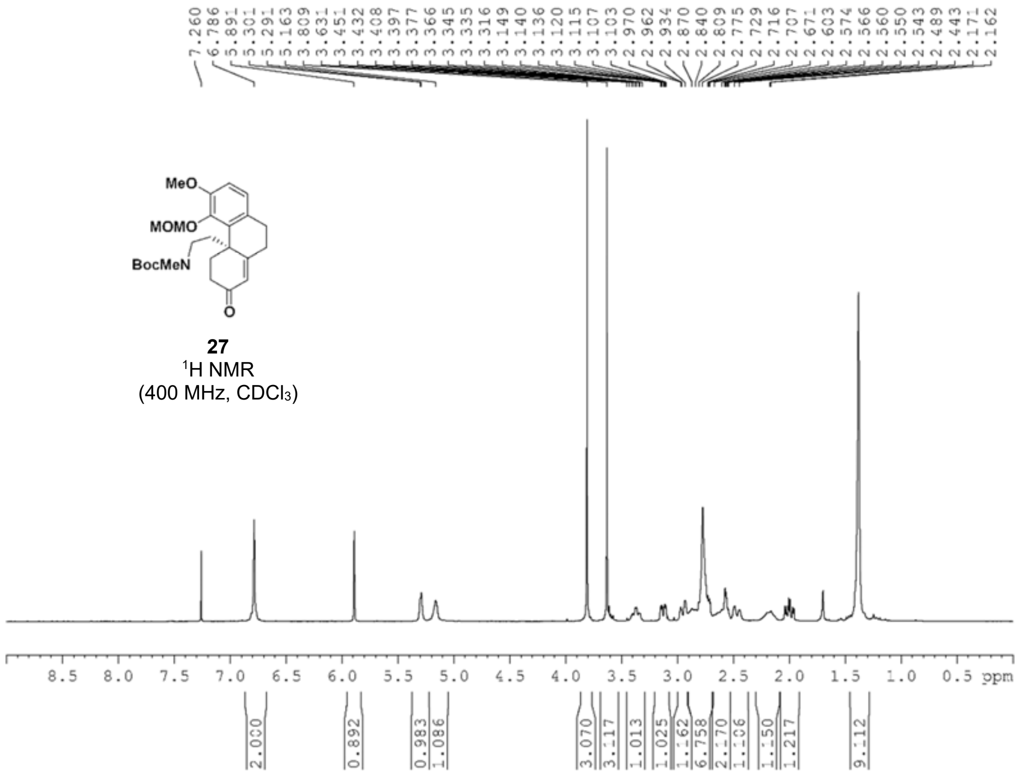

Supplementary Figure 25. ^{1}H NMR spectrum of **27**

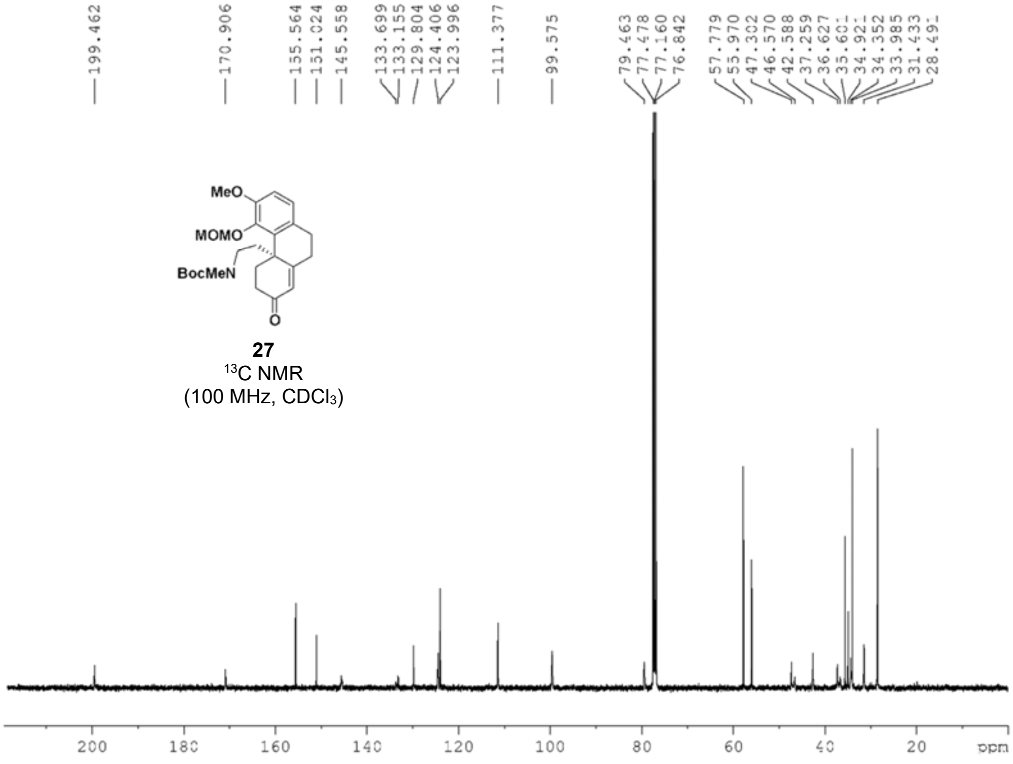

Supplementary Figure 26. ^{13}C NMR spectrum of **27**

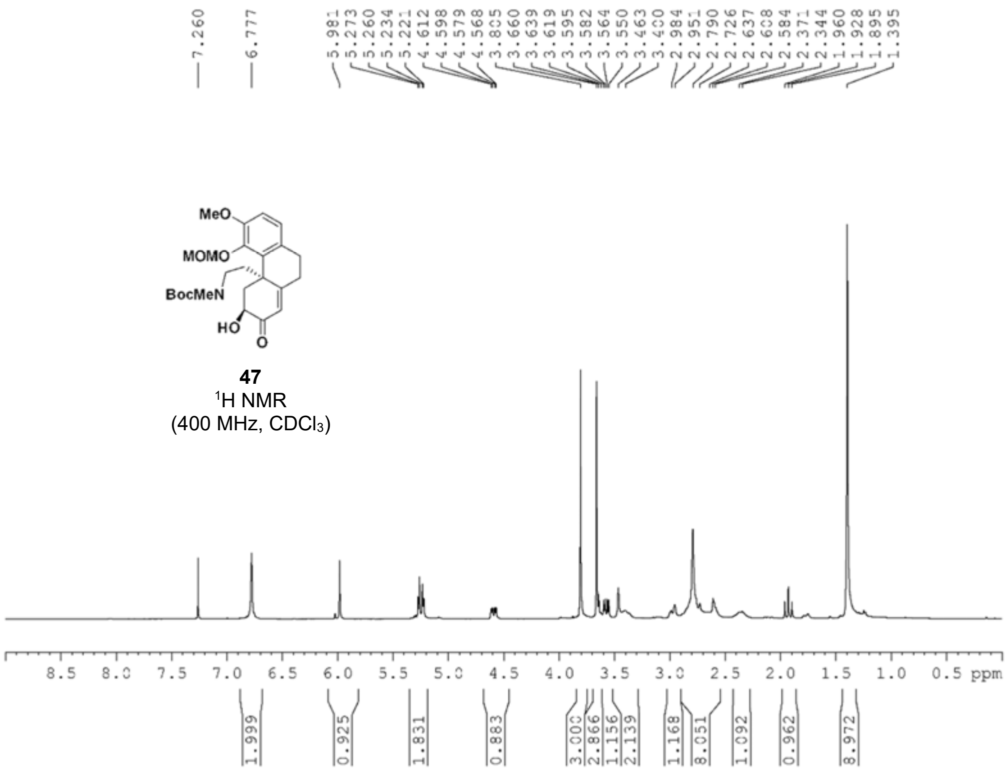

Supplementary Figure 27. ^{1}H NMR spectrum of **47**

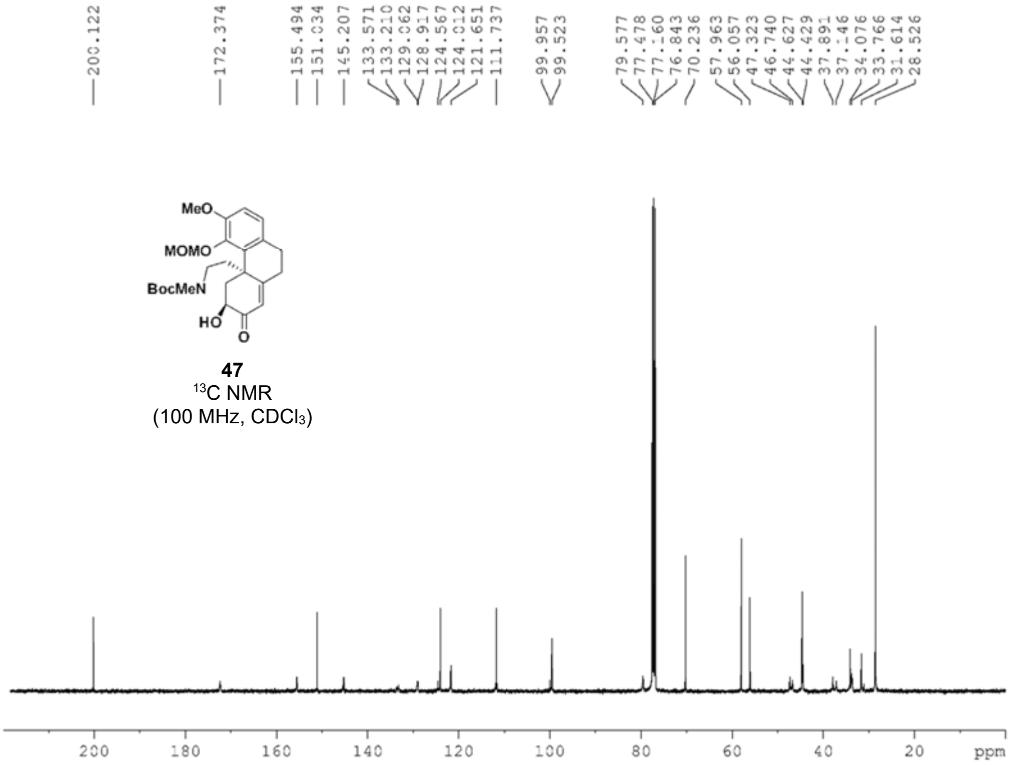

Supplementary Figure 28. ^{13}C NMR spectrum of **47**

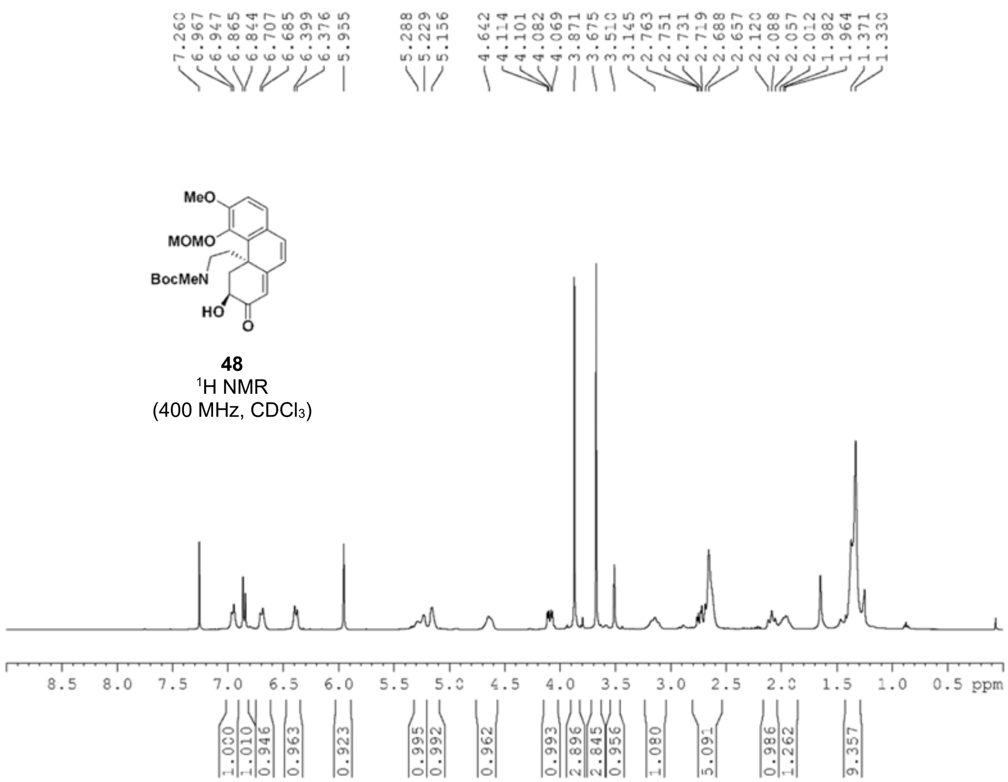

Supplementary Figure 29. ^{1}H NMR spectrum of **48**

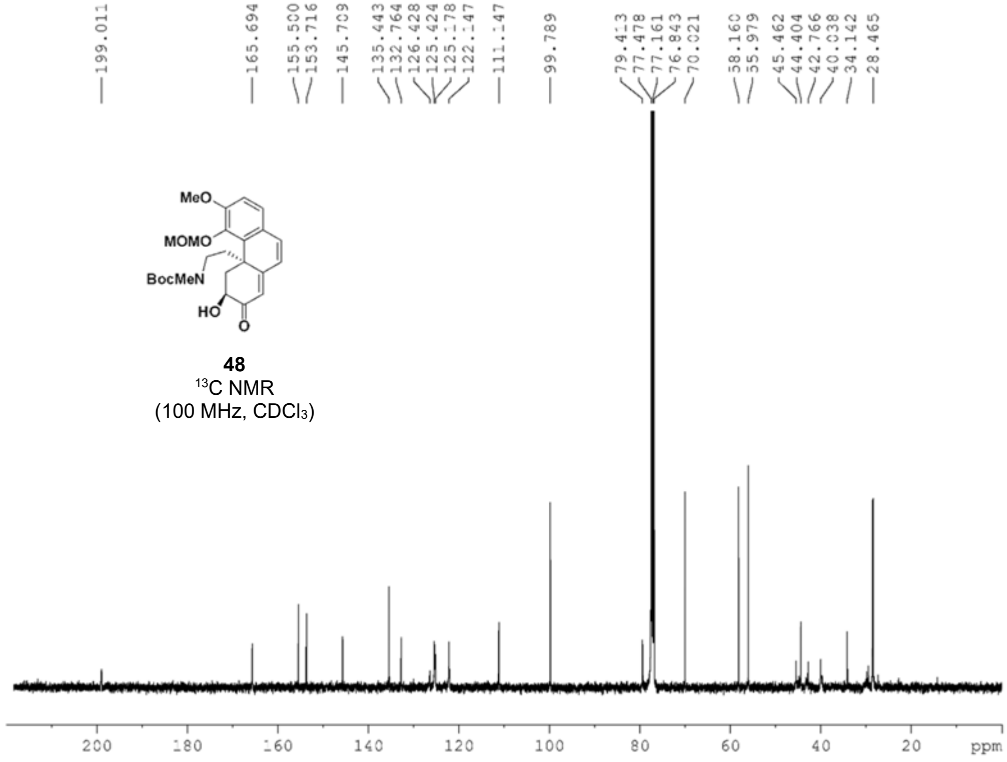

Supplementary Figure 30. ^{13}C NMR spectrum of **48**

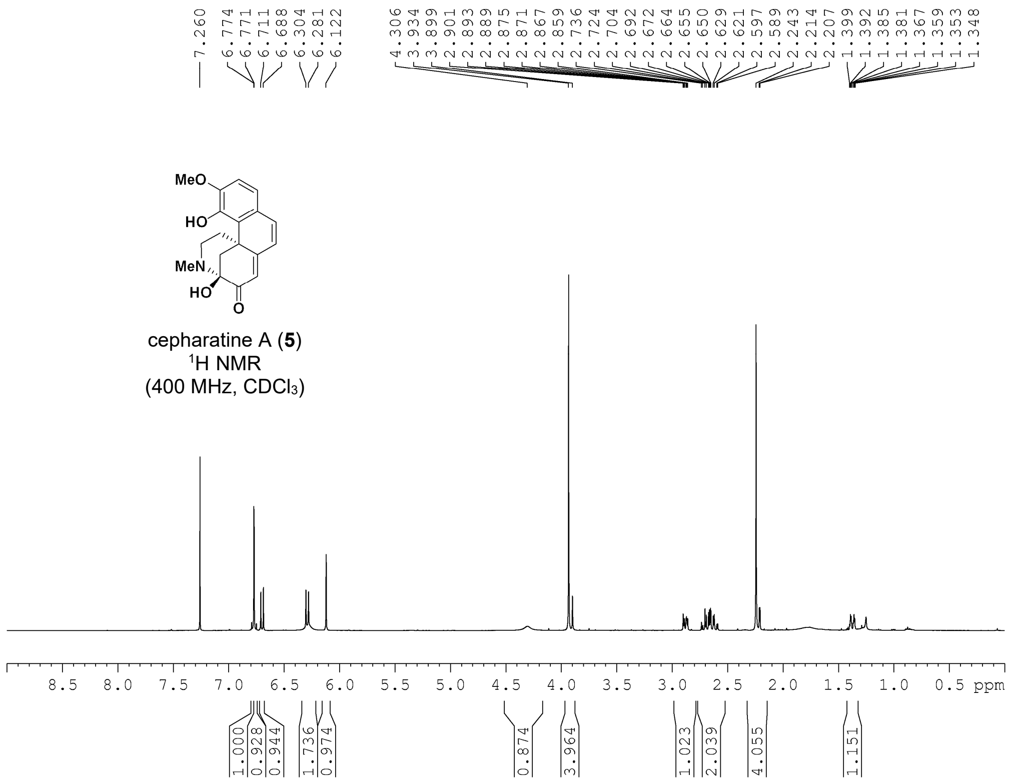

Supplementary Figure 31. ^{1}H NMR spectrum of cepharatine A (**5**)

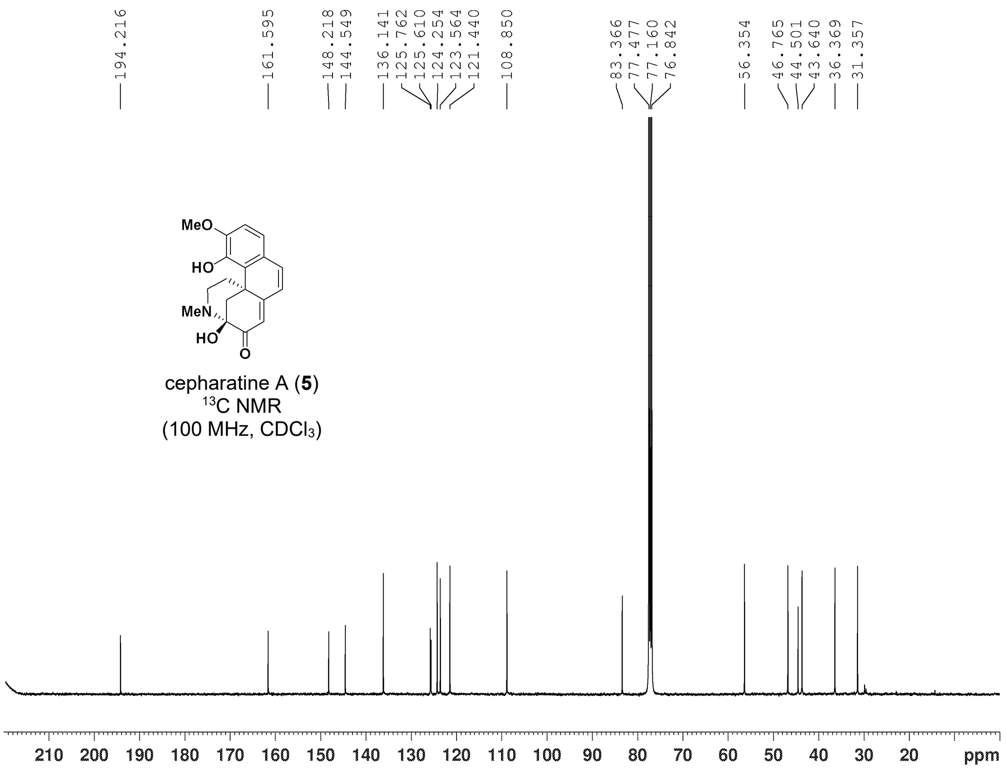

Supplementary Figure 32. ^{13}C NMR spectrum of cepharatine A (**5**)

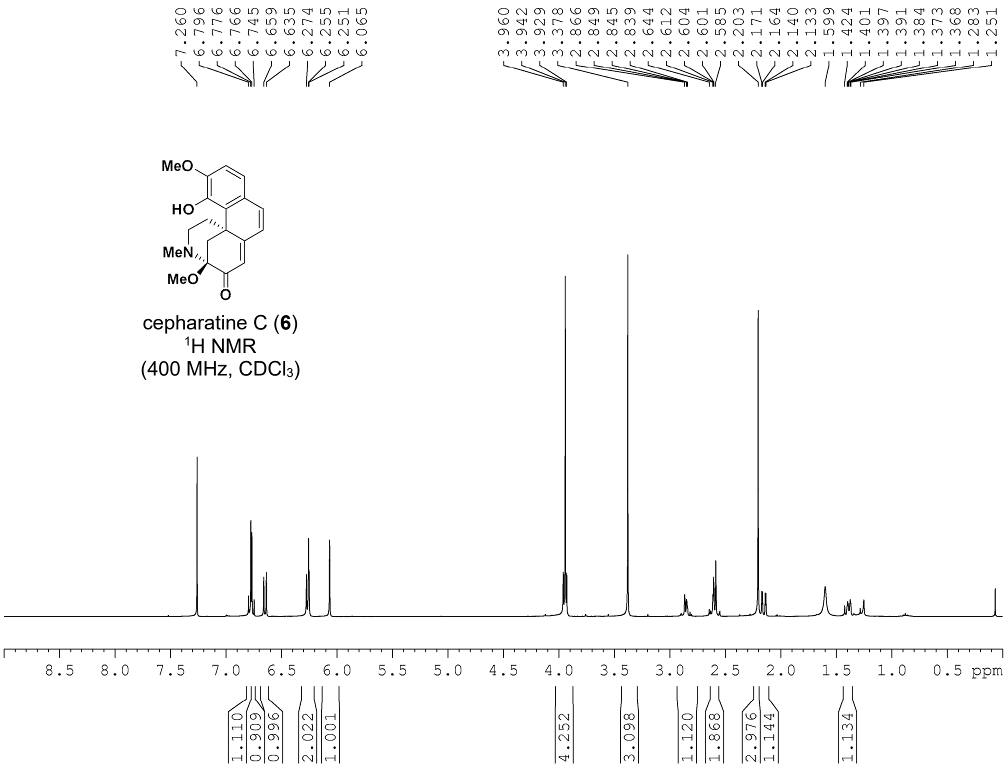

Supplementary Figure 33. ^{1}H NMR spectrum of cepharatine C (**6**)

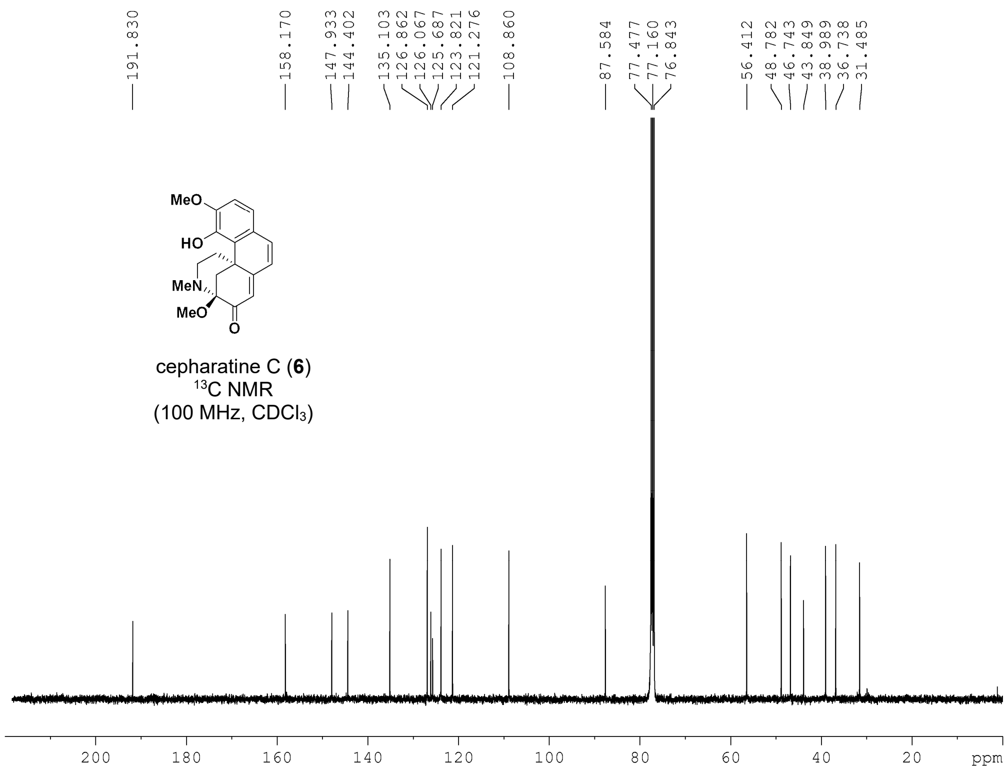

Supplementary Figure 34. ^{13}C NMR spectrum of cepharatine C (**6**)

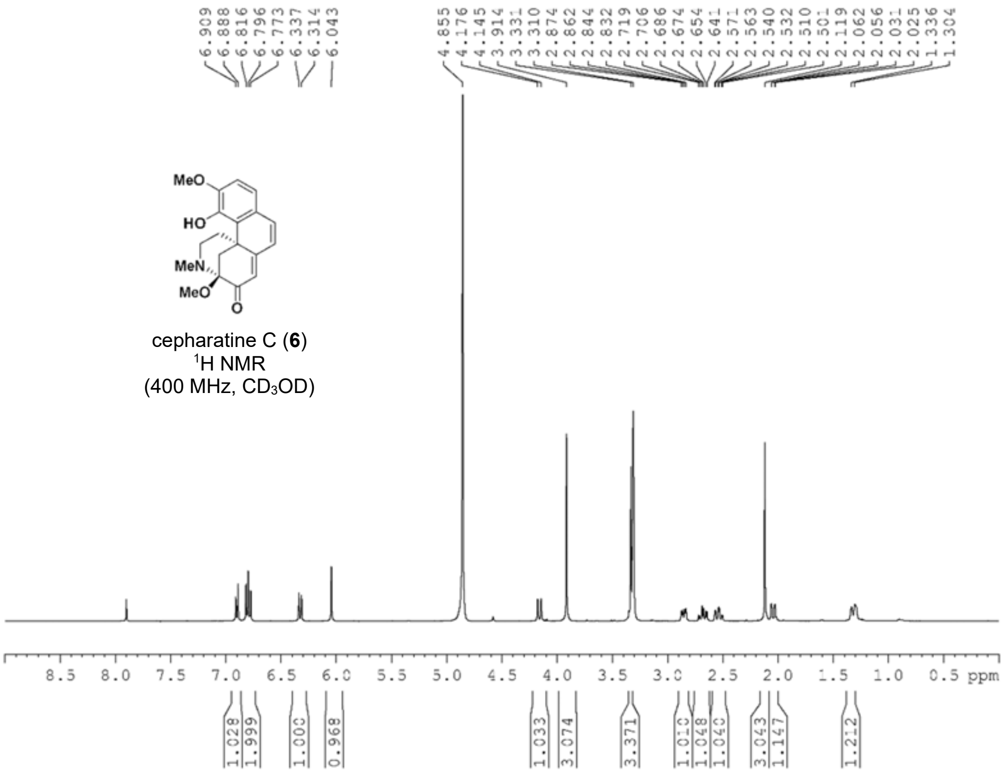

Supplementary Figure 35. ^{1}H NMR spectrum of cepharatine C (**6**) (in CD_3OD)

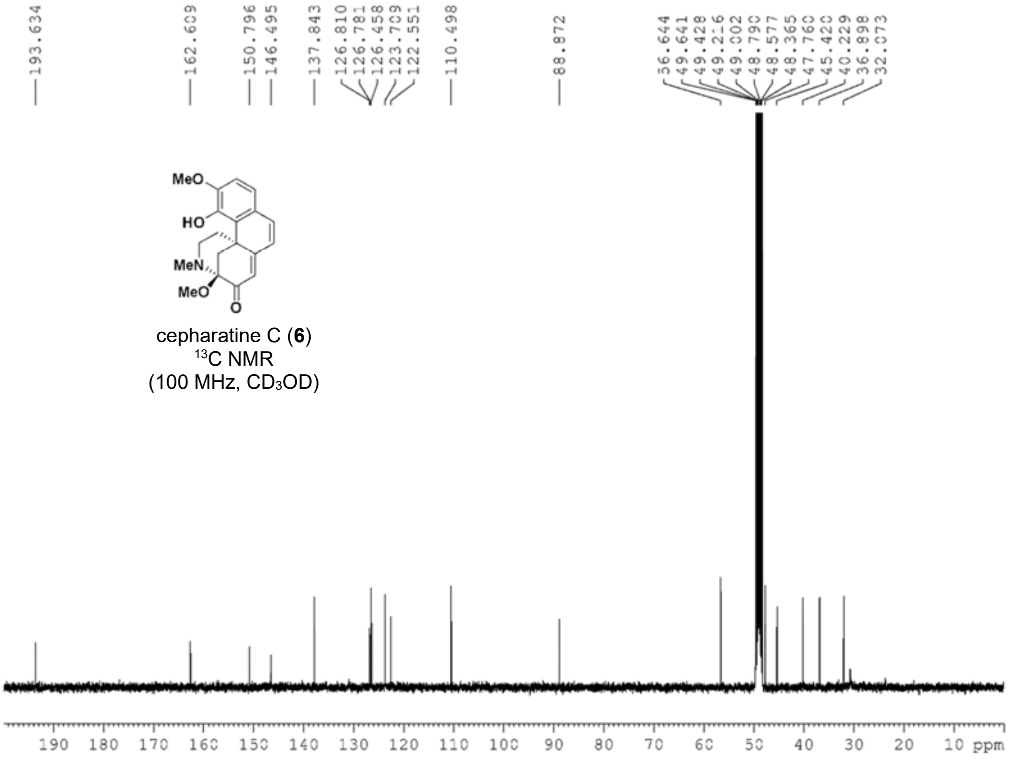

Supplementary Figure 36. ^{13}C NMR spectrum of cepharatine C (**6**) (in CD_3OD)

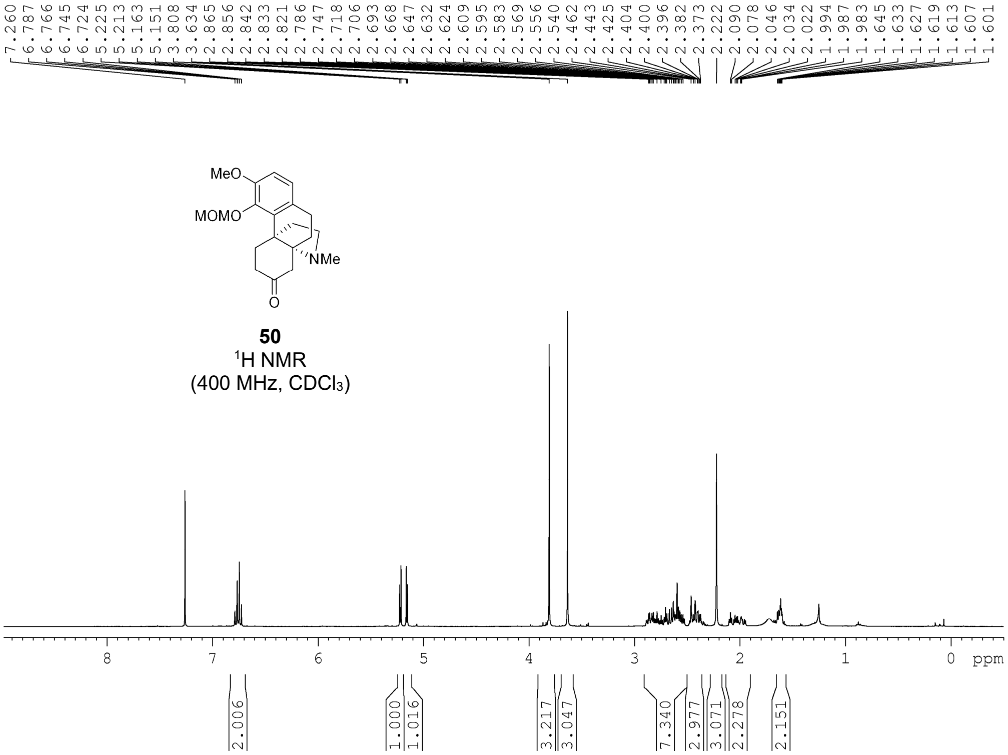

Supplementary Figure 37. ^{1}H NMR spectrum of **50**

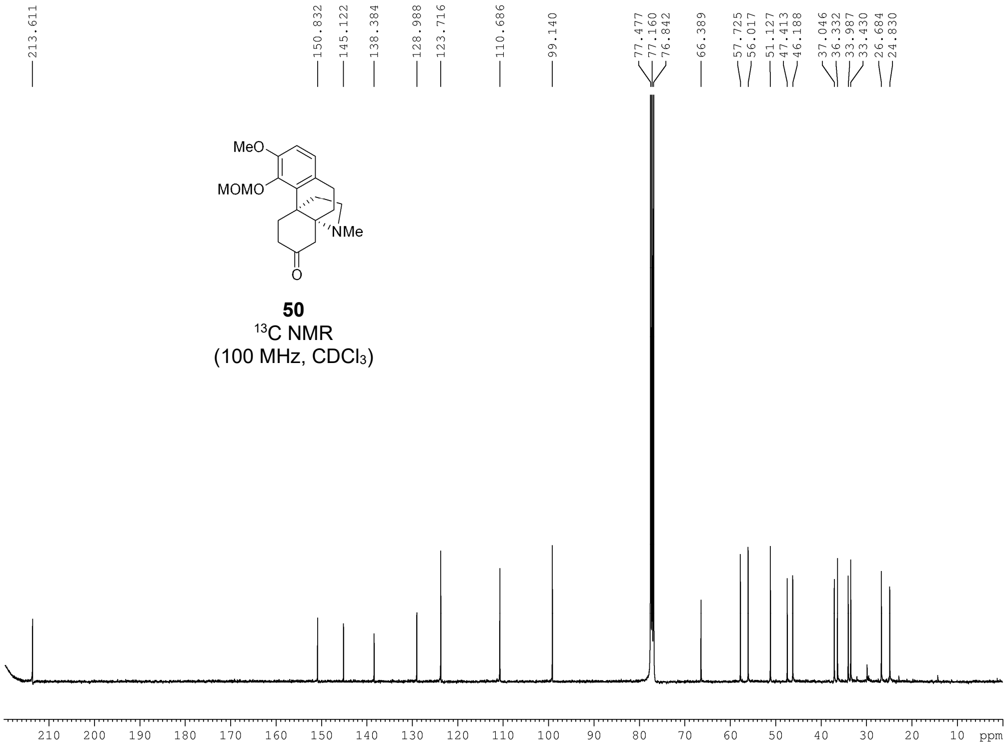

Supplementary Figure 38. ^{13}C NMR spectrum of **50**

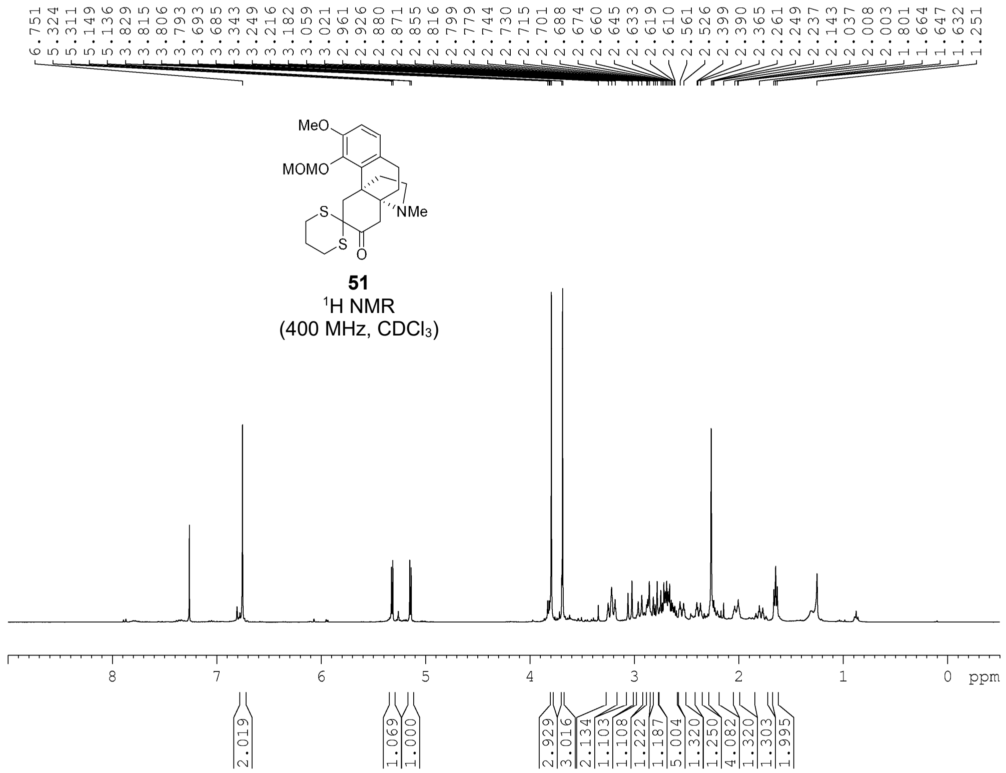

Supplementary Figure 39. ^{1}H NMR spectrum of **51**

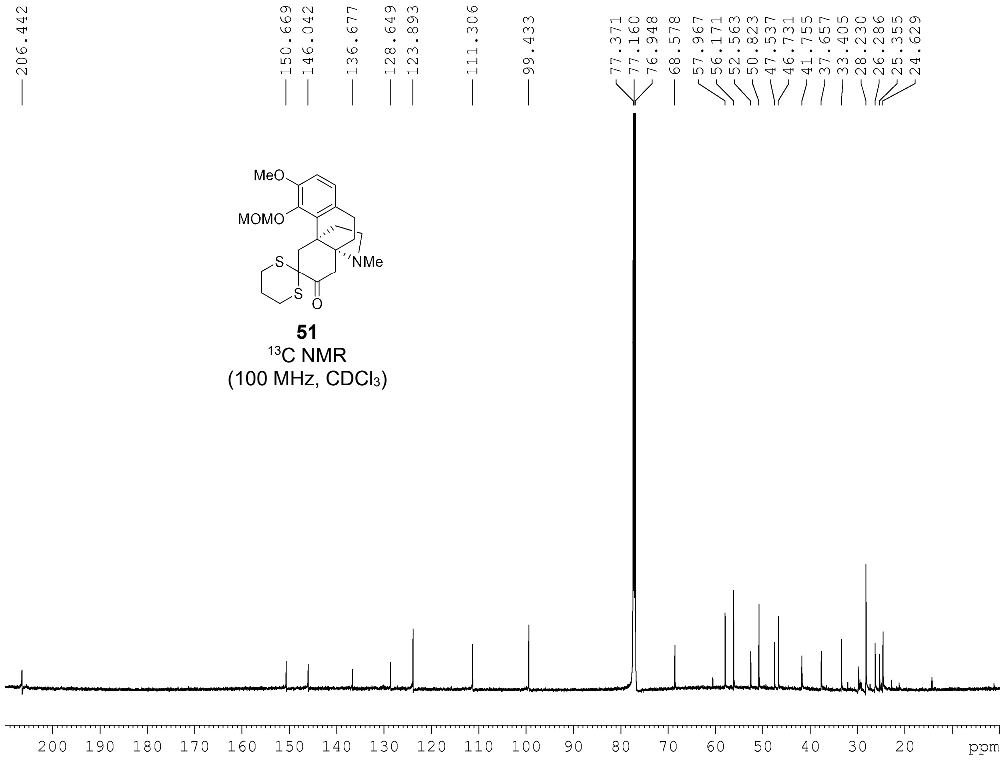

Supplementary Figure 40. ^{13}C NMR spectrum of **51**

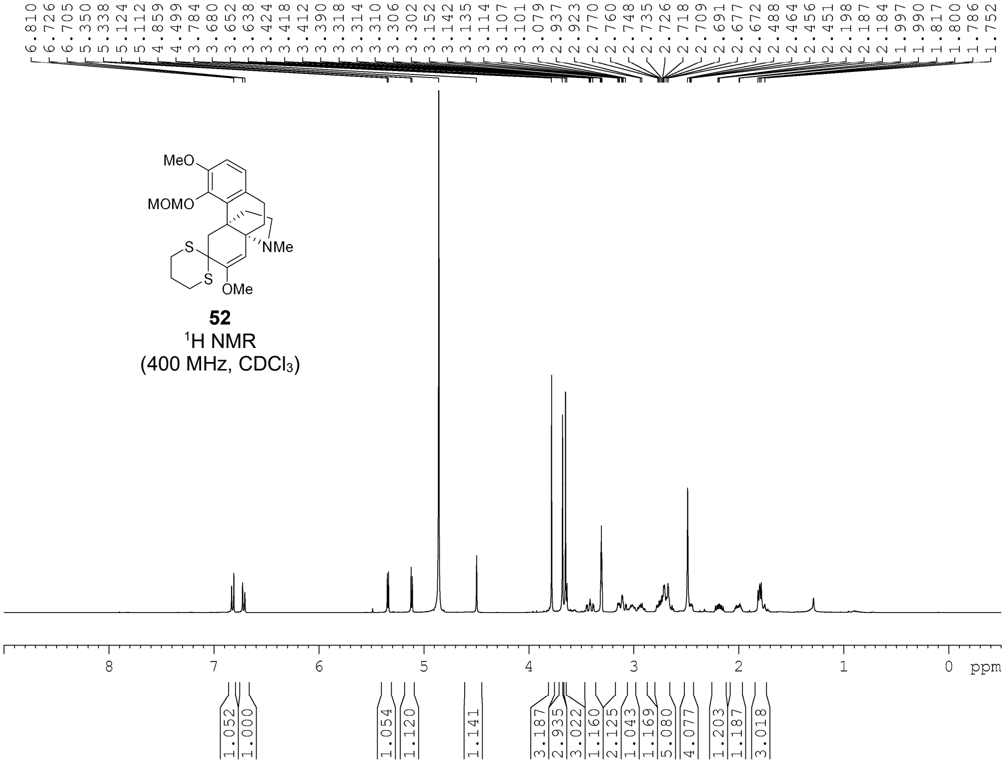

Supplementary Figure 41. ^{1}H NMR spectrum of **52**

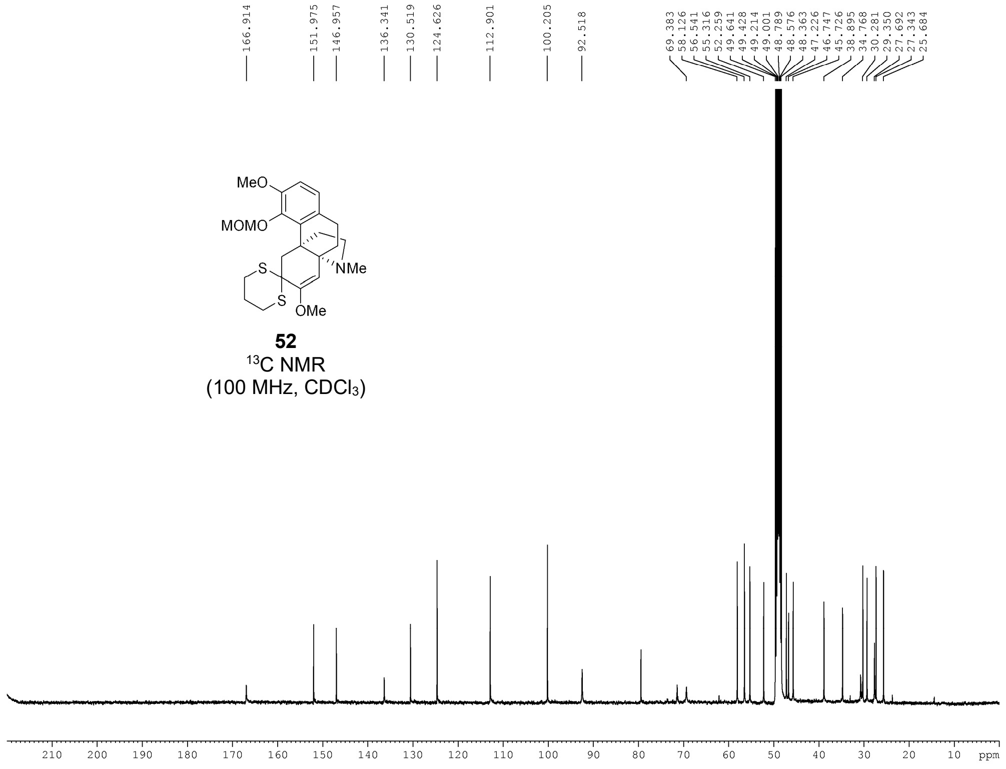

Supplementary Figure 42. ^{13}C NMR spectrum of **52**

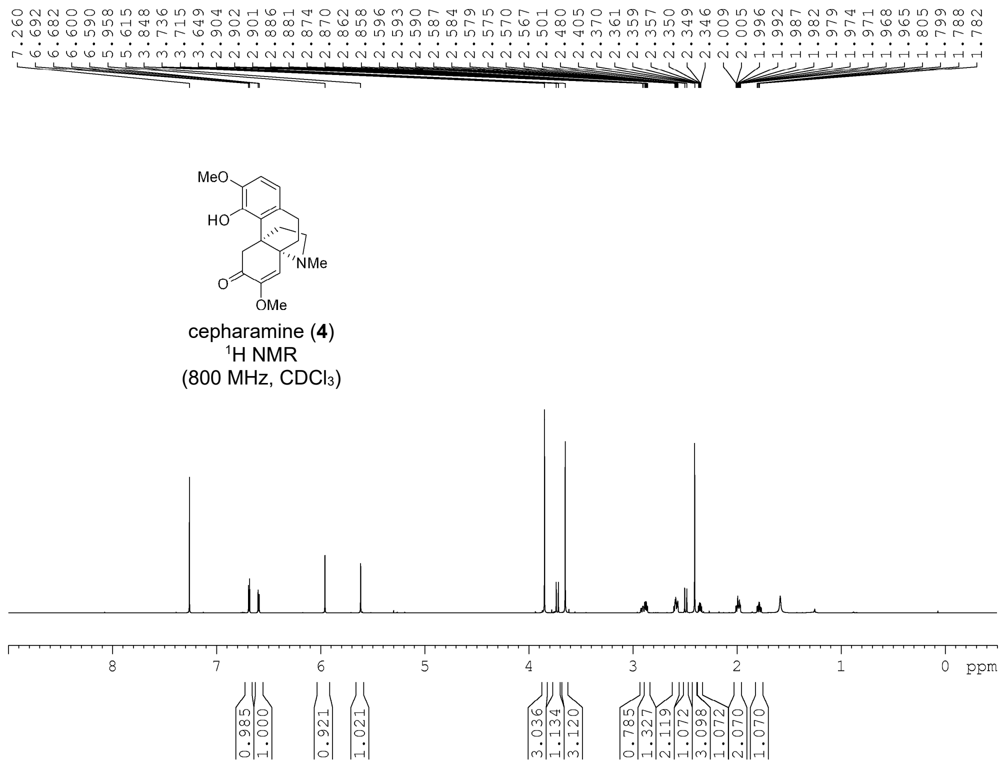

Supplementary Figure 43. ^{1}H NMR spectrum of cepharamine (**4**)

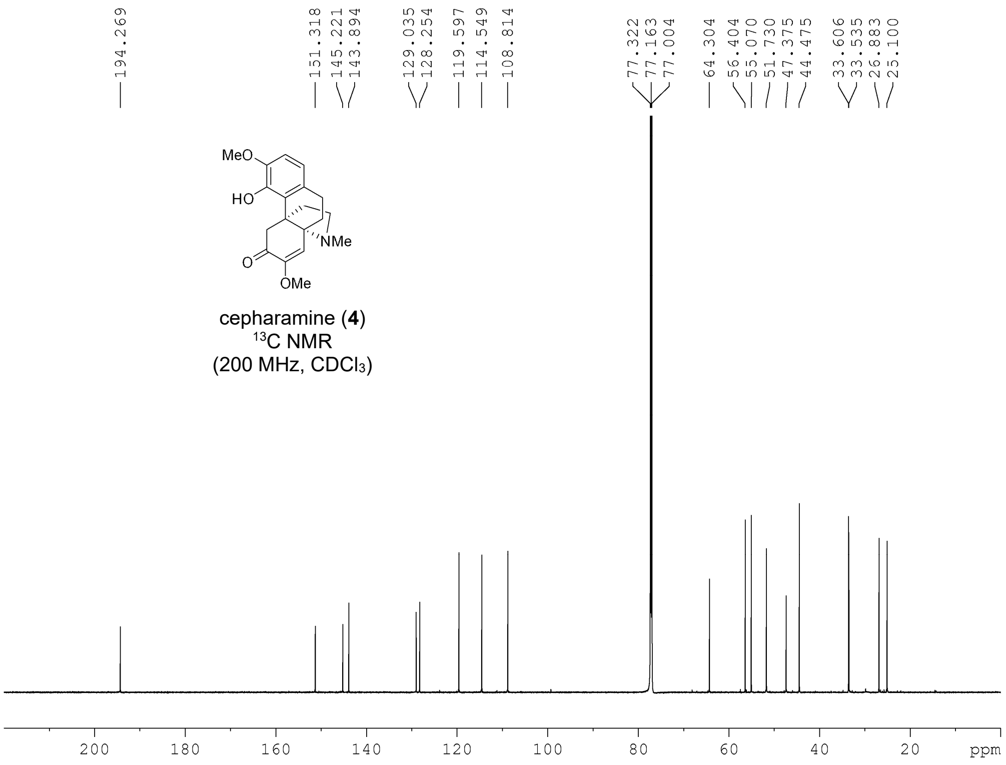

Supplementary Figure 44. ^{13}C NMR spectrum of cepharamine (**4**)

Supplementary Tables

Supplementary table1: Chemical Shifts of ^{13}C NMR for the Natural and the Synthetic sinoracutine in $CDCl_3$

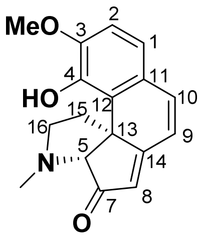

sinoracutine

Carbon	Natural[1] ($CDCl_3$)	Synthetic ($CDCl_3$)
1	121.24	121.3
2	109.42	109.5
3	151.07	151.2
4	145.58	145.7
5	72.31	72.4
7	206.56	206.8
8	123.27	123.4
9	118.00	118.1
10	138.10	138.3
11	124.11	124.2
12	127.59	127.7
13	53.71	53.8
14	174.94	175.0
15	41.52	41.7
16	51.46	51.5
3-OCH_3	55.87	56.0
N-CH_3	36.35	36.4

[a] The difference in chemical shifts can be explained by the calibration of the $CDCl_3$ at 77.0 ppm in ref 1. In our case, $CDCl_3$ was calibrated at 77.16 ppm according to the literature (Gottlieb, H., Kotlyar, V. & Nudelman, A. *J. Org. Chem*. **62**, 7512 (1997).)

Supplementary table 2: Chemical Shifts of ^{13}C NMR for the Natural and the Synthetic cepharatine A in $CDCl_3$

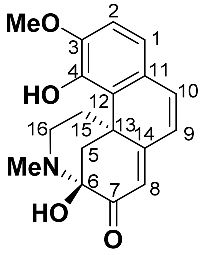

cepharatine A

Carbon	Natural[2] ($CDCl_3$)	Synthetic ($CDCl_3$)
1	121.2	121.4
2	108.7	108.8
3	148.1	148.2
4	144.4	144.5
5	43.4	43.6
6	83.2	83.4
7	194.0	194.2
8	124.0	124.3
9	123.3	123.6
10	136.1	136.1
11	125.5	125.8
12	125.4	125.6
13	44.3	44.5
14	161.6	161.6
15	31.1	31.4
16	46.6	46.8
3-OMe	56.1	56.4
N-Me	36.2	36.4

[a] The difference in chemical shifts can be explained by the calibration of the $CDCl_3$ at 77.0 ppm in ref 2. In our case, $CDCl_3$ was calibrated at 77.16 ppm according to the literature (Gottlieb, H., Kotlyar, V. & Nudelman, A. *J. Org. Chem*. **62**, 7512 (1997).)

Supplementary table 3: Chemical Shifts of ^{13}C NMR for the Natural and the Synthetic cepharatine C in CD_3OD

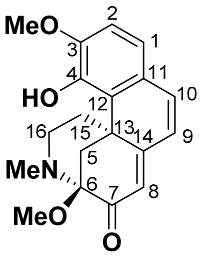

cepharatine C

Carbon	Natural[2] (CD_3OD)	Synthetic (CD_3OD)
1	122.6	122.6
2	110.4	110.5
3	150.8	150.8
4	146.5	146.5
5	40.1	40.2
6	88.8	88.9
7	193.6	193.6
8	126.4	126.5
9	123.7	123.7
10	137.9	137.8
11	126.8	126.81
12	126.7	126.78
13	45.4	45.4
14	162.7	162.6
15	32.0	32.1
16	47.7	47.8
3-OMe	56.6	56.6
N-Me	36.9	36.9

Supplementary table 4: Chemical Shifts of ^{13}C NMR for the Natural and the Synthetic cepharamine in $CDCl_3$

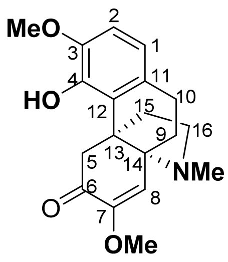

cepharamine

Carbon	Natural[3] (800 MHz, $CDCl_3$)	Synthetic (800 MHz, $CDCl_3$)
1	119.44	119.6
2	108.69	108.8
3	145.08	145.2
4	143.75	143.9
5	44.34	44.5
6	194.12	194.3
7	151.17	151.3
8	114.43	114.5
9	26.73	26.9
10	24.96	25.1
11	128.89	129.0
12	128.11	128.3
13	47.23	47.4
14	64.16	64.3
15	33.46	33.6
16	51.58	51.7
3-OMe	56.26	56.4
7-OMe	54.93	55.1
N-Me	33.39	33.5

[a] The difference in chemical shifts can be explained by the calibration of the $CDCl_3$ at 77.0 ppm in ref 3. In our case, $CDCl_3$ was calibrated at 77.16 ppm according to the literature (Gottlieb, H., Kotlyar, V. & Nudelman, A. *J. Org. Chem*. **62**, 7512 (1997).)

Supplementary table 5: Copy of HPLC chromatogram of **28**

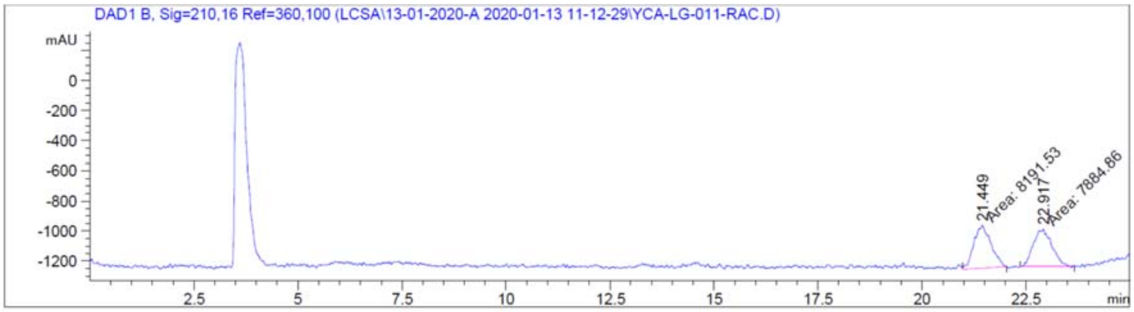

Signal 2: DAD1 B, Sig=210,16 Ref=360,100

Peak #	RetTime [min]	Type	Width [min]	Area [mAU*s]	Height [mAU]	Area %
1	21.449	MM	0.4770	8191.53174	286.20956	50.9538
2	22.917	MM	0.5259	7884.86328	249.86201	49.0462
Totals :				1.60764e4	536.07158	

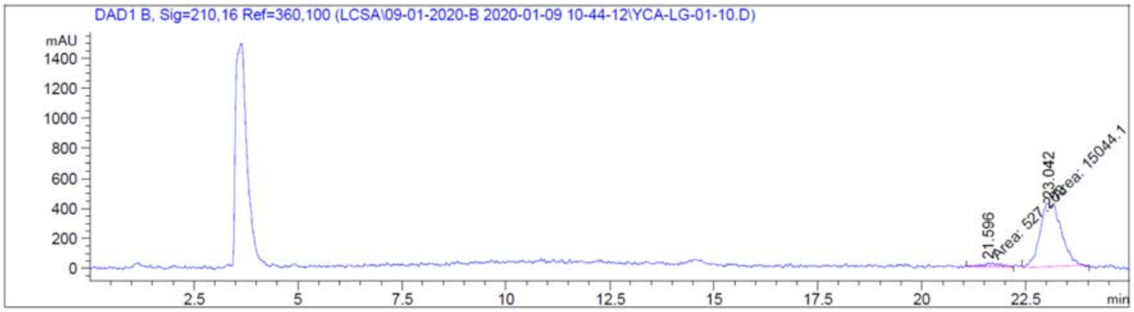

Signal 2: DAD1 B, Sig=210,16 Ref=360,100

Peak #	RetTime [min]	Type	Width [min]	Area [mAU*s]	Height [mAU]	Area %
1	21.596	MM	0.3857	527.20294	22.77886	3.3857
2	23.042	MM	0.5881	1.50441e4	426.34012	96.6143
Totals :				1.55713e4	449.11898	

HPLC: ID column, Hexane/IPA = 80/20, 1.0 mL/min, λ = 210 nm, tR (minor) = 21.60 min, tR (major) = 23.04 min.

Supplementary table 6: Copy of HPLC chromatogram of (-)-sinoracutine (**9**)

Additional Info : Peak(s) manually integrated

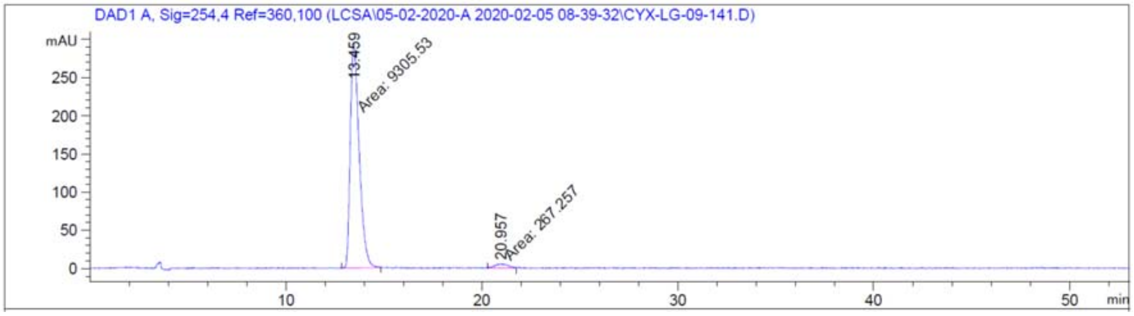

Signal 1: DAD1 A, Sig=254,4 Ref=360,100

Peak #	RetTime [min]	Type	Width [min]	Area [mAU*s]	Height [mAU]	Area %
1	13.459	MM	0.5272	9305.52734	294.20871	97.2082
2	20.957	MM	0.7988	267.25714	5.57588	2.7918
Totals :				9572.78448	299.78459	

Supplementary Methods

General Information.

General Analytical Information.

NMR spectra were recorded on a Brüker AvanceIII-400, Brüker Avance-400 or Brüker DPX-400 spectrometer at room temperature, ^{1}H frequency is at 400.13 MHz, ^{13}C frequency is at 100.62 MHz. Chemical shifts (δ) were reported in parts per million (ppm) relative to residual solvent peaks rounded to the nearest 0.01 for proton and 0.1 for carbon (*ref: $CHCl_3$ [^{1}H: 7.26, ^{13}C: 77.16]*. Coupling constants (J) were reported in Hz to the nearest 0.1 Hz. Peak multiplicity was indicated as follows s (singlet), d (doublet), t (triplet), q (quartet), m (multiplet) and br (broad). Attribution of peaks was done using the multiplicities and integrals of the peaks.

IR spectra were recorded in a Jasco FT/IR-4100 spectrometer outfitted with a PIKE technology MIRacleTM ATR accessory as neat films compressed onto a Zinc Selenide window. The spectra were reported in cm^{-1}.

The accurate masses were measured by the mass spectrometry service of the EPFL by ESI-TOF using a QTOF Ultima from Waters or APPI-FT-ICR using a linear ion trap Fourier transform ion cyclotron resonance mass spectrometer from Thermo Scientific.

Melting points were measured using a Stuart SMP30

Materials and Methods.

Unless otherwise stated, starting materials were purchased from Aldrich and/or Fluka. Solvents were purchased in HPLC quality, degassed by purging thoroughly with nitrogen and dried over activated molecular sieves of appropriate size. Alternatively, they were purged with argon and passed through alumina columns in a solvent purification system (Innovative Technology). Conversion was monitored by thin layer chromatography (TLC) using Merck TLC silica gel 60 F254. Compounds were visualized by UV light at 254 nm and by dipping the plates in an ethanolic vanillin/sulfuric acid solution or an aqueous potassium permanganate solution followed by heating. Flash column chromatography was performed over silica gel (230–400 mesh).

Experimental Procedures and Characterization Data

Synthesis of compound 32

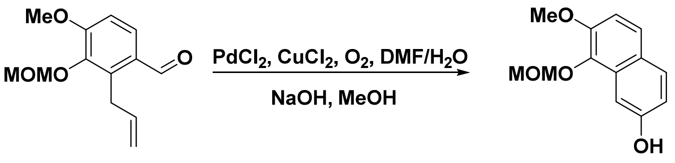

7-methoxy-8-(methoxymethoxy)naphthalen-2-ol:[4] To a solution of **31** (11.8 g, 50.0 mmol, 1.0 equiv) in DMF/H_2O (7:1, 70/10 mL) were added $PdCl_2$ (443 mg, 2.5 mmol, 0.05 equiv) and $CuCl_2$ (10.08 g, 75.0 mmol, 1.5 equiv) sequentially at room temperature. The reaction mixture was bubbled with oxygen for 2 h and stirred overnight. MeOH (80 mL) and NaOH (8.0 g, 200.0 mmol, 4.0 equiv) were added to the reaction mixture and the mixture was stirred at room temperature for 2 h. The residue was diluted with water (200 mL) and extracted with ethyl acetate. The combined organic extracts were washed with brine, dried over Na_2SO_4, filtered and concentrated *in vacuo*. The crude product was purified by flash column chromatography (SiO_2, PE/AcOEt 5:1) to yield the pure product **32** (8.5 g, 73%) as a yellow oil.

^{1}H NMR (400 MHz, $CDCl_3$): δ 7.65 (d, *J* = 8.9 Hz, 1H), 7.53 (d, *J* = 9.0 Hz, 1H), 7.52 (br s, 1H), 7.11 (d, *J* = 8.9 Hz, 1H), 6.98 (dd, *J* = 8.8, 2.5 Hz, 1H), 6.47 (br, 1H), 5.28 (s, 2H), 3.95 (s, 3H), 3.64 (s, 3H).

^{13}C NMR (100 MHz, $CDCl_3$): δ 154.7, 148.7, 138.3, 130.7, 129.9, 125.3, 124.8, 116.5, 112.1, 103.1, 99.2, 58.0, 56.8.

IR: ν (cm^{-1}) 3361 (w), 1630 (s), 1514 (m), 1479 (m), 1450 (m), 1369 (m), 1257 (s), 1215 (s), 1153 (s), 1072 (s), 1039 (s), 964 (s), 825 (s), 789 (s), 721 (s).

HRMS: (APCI/QTOF) m/z: $[M + Na]^+$ Calcd for $C_{13}H_{14}NaO_4^+$ 257.0784; Found 257.0790.

Synthesis of compound 33

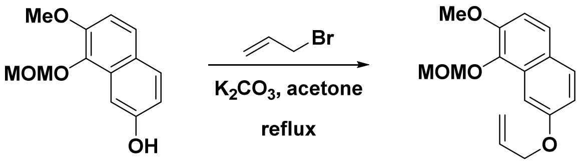

7-(allyloxy)-2-methoxy-1-(methoxymethoxy)naphthalene:[5] Under N_2 atmosphere, to a solution of compound **32** (8.2 g, 35.0 mmol, 1.0 equiv) in acetone (100 mL) was added K_2CO_3 (9.7 g, 70.0 mmol, 2.0 equiv) and allyl bromide (4.5 mL, 52.5 mmol, 1.5 equiv) sequentially. The reaction mixture was

heated to reflux and stirred at that temperature overnight. The reaction mixture was cooled to room temperature and diluted with diethyl ether (200 mL), filtered and concentrated *in vacuo*. The crude product was purified by flash column chromatography (SiO_2, PE/EtOAc 10:1) to yield the pure product **33** (9.4 g, 98%) as a yellow oil.

^{1}H NMR (400 MHz, CDCl$_3$): δ 7.67 (d, *J* = 9.0 Hz, 1H), 7.54 (d, *J* = 9.0 Hz, 1H), 7.49 (d, *J* = 2.3 Hz, 1H), 7.13 (d, *J* = 8.9 Hz, 1H), 7.06 (dd, *J* = 9.0, 2.5 Hz, 1H), 6.20-6.09 (m, 1H), 5.49 (dq, *J* = 19.0, 5.0, 3.2, 1.8 Hz, 1H), 5.33 (dq, *J* = 12.0, 3.9, 2.6, 1.4 Hz, 1H), 5.29 (s, 2H), 4.69 (t, *J* = 1.4 Hz, 1H), 4.68 (t, *J* = 1.4 Hz, 1H), 3.95 (s, 3H), 3.66 (s, 3H).

^{13}C NMR (100 MHz, CDCl$_3$): δ 157.2, 148.7, 139.0, 133.3, 130.6, 129.4, 125.4, 124.4, 117.9, 117.5, 112.2, 100.7, 99.2, 68.8, 57.9, 56.8.

IR: ν (cm^{-1}) 2839 (w), 2362 (m), 1628 (s), 1512 (s), 1446 (m), 1360 (m), 1261 (s), 1215 (s), 1155 (s), 1072 (s), 1043 (s), 957 (s), 928 (s), 825 (s), 715 (m), 673 (m).

HRMS: (ESI/QTOF) m/z: $[M + Na]^+$ Calcd for $C_{16}H_{18}NaO_4^+$ 297.1097; Found 297.1102.

Synthesis of compound 29

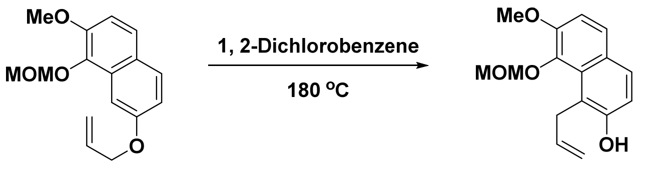

33 29

1-allyl-7-methoxy-8-(methoxymethoxy)naphthalen-2-ol:[6] Under N_2 atmosphere, a solution of compound **33** (4.1 g, 15.0 mmol, 1.0 equiv) in 1,2-dichlorobenzene (10 mL) was heated to 180 °C and stirred at that temperature for 5 h. The reaction mixture was cooled to room temperature and purified by flash column chromatography (SiO_2, PE/EtOAc 1:0 to 10:1) to yield the pure product **29** (3.5 g, 86%) as a yellow oil.

^{1}H NMR (400 MHz, CDCl$_3$): δ 7.57 (d, *J* = 8.8 Hz, 1H), 7.53 (d, *J* = 8.9 Hz, 1H), 7.13 (d, *J* = 9.0 Hz, 1H), 6.98 (d, *J* = 8.7 Hz, 1H), 6.29-6.17 (m, 1H), 5.72 (br, 1H), 5.16 (s, 2H), 5.15-5.06 (m, 2H), 4.29 (td, *J* = 5.6, 1.7 Hz, 2H), 3.94 (s, 3H), 3.62 (s, 3H).

^{13}C NMR (100 MHz, CDCl$_3$): δ 153.7, 150.2, 140.4, 137.6, 128.9, 128.8, 126.8, 125.8, 116.6, 115.3, 115.1, 111.8, 100.0, 58.1, 56.7, 31.1.

IR: ν (cm^{-1}) 3383 (w), 2940 (w), 1622 (m), 1514 (s), 1459 (m), 1436 (m), 1348 (m), 1264 (s), 1152 (m), 1132 (s), 1076 (m), 1015 (s), 987 (s), 914 (s), 822 (s), 718 (s)

HRMS: (APPI/LTQ-Orbitrap) m/z: $[M]^+$ Calcd for $C_{16}H_{18}O_4^+$ 274.1200; Found 274.1202.

Synthesis of compound 28

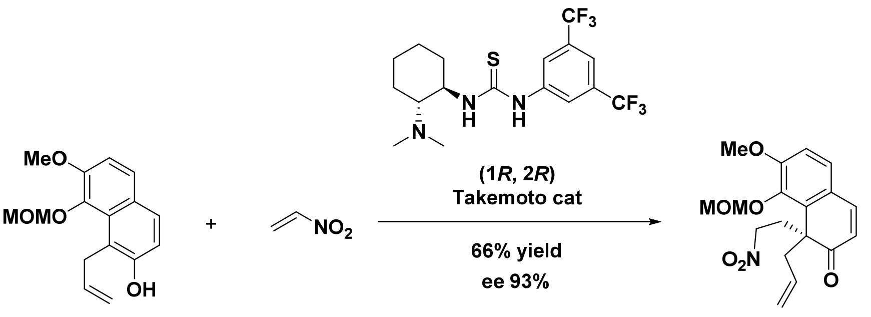

29 30 28

(*R*)-1-allyl-7-methoxy-8-(methoxymethoxy)-1-(2-nitroethyl)naphthalen-2(1H)-one:[7] Under N_2 atmosphere, to a solution of compound **29** (2.74 g, 10.0 mmol, 1.0 equiv) and 3Å MS (250 mg) in DCM (50 mL) was added the catalyst (207 mg, 0.05 mmol, 0.05 equiv) and the mixture was stirred for 10 min, then a solution of nitroethylene (**30**) (10.0 mL, 20.0 mmol, 2.0 equiv, 2 M in toluene) was added and the mixture was stirred at room temperature overnight. The reaction mixture was quenched with saturated $NaHCO_3$ and extracted with DCM. The combined organic extracts were washed with brine, dried over Na_2SO_4, filtered and concentrated *in vacuo*. The crude product was purified by flash column chromatography (SiO_2, PE/AcOEt 3:1) to yield the pure product **28** (2.3 g, 66%) as a yellow oil.

^{1}H NMR (400 MHz, $CDCl_3$): δ 7.35 (d, *J* = 9.8 Hz, 1H), 7.06 (d, *J* = 8.4 Hz, 1H), 6.93 (d, *J* = 8.4 Hz, 1H), 6.10 (d, *J* = 9.7 Hz, 1H), 5.31 (d, *J* = 5.2 Hz, 1H), 5.29-5.18 (m, 1H), 5.21 (d, *J* = 5.2 Hz, 1H), 4.89-4.83 (m, 1H), 4.77-4.73 (m, 1H), 4.03-3.85 (m, 2H), 3.90 (s, 3H), 3.63 (s, 3H), 3.38-3.25 (m, 2H), 2.88 (ddd, *J* = 13.2, 10.4, 6.0 Hz, 1H), 2.75 (ddt, *J* = 13.2, 7.5, 1.1 Hz, 1H).

^{13}C NMR (100 MHz, $CDCl_3$): δ 202.2, 154.0, 146.6, 145.9, 133.0, 132.5, 126.7, 125.2, 123.8, 118.2, 111.6, 99.8, 72.1, 58.0, 56.0, 53.8, 44.8, 35.0.

IR: ν (cm^{-1}) 2021 (m), 1649 (s), 1591 (s), 1552 (s), 1435 (s), 1271 (s), 1165 (s), 1039 (s), 920 (s), 719 (s).

$[\alpha]_D^{24}$ -10.0 (c 1.06, $CHCl_3$).

HPLC: 93% ee, ID column, Hexane/IPA = 80/20, 1.0 mL/min, λ = 210 nm, tR (minor) = 21.6 min, tR (major) = 23.0 min.

HRMS: (ESI/QTOF) m/z: $[M + Na]^+$ Calcd for $C_{18}H_{21}NNaO_6^+$ 370.1261; Found 370.1266.

Synthesis of compound 35

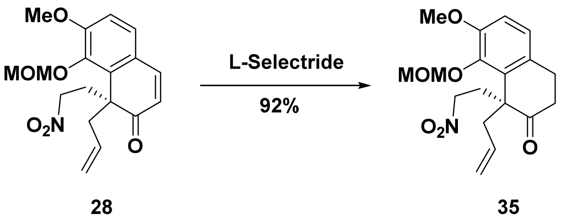

(*R*)-1-allyl-7-methoxy-8-(methoxymethoxy)-1-(2-nitroethyl)-3,4-dihydronaphthalen-2(1H)-one:[7]
Under N_2 atmosphere, to a solution of compound **28** (2.0 g, 5.8. mmol, 1.0 equiv) in dry THF (50 mL) was added L-Selectride (7.0 mL, 7.0 mmol, 1.0 M in THF, 1.2 equiv) at -78 ºC and the mixture was stirred at that temperature for 2 h. The reaction mixture was quenched with saturated NH_4Cl and extracted with ethyl acetate. The combined organic extracts were washed with brine, dried over Na_2SO_4, filtered and concentrated *in vacuo*. The crude product was purified by flash column chromatography (SiO_2, PE/AcOEt 4:1) to yield the pure product **35** (1.86 g, 92%) as a yellow oil.

^{1}H NMR (400 MHz, $CDCl_3$): δ 6.85 (br s, 2H), 5.50-5.41 (m, 1H), 5.27 (d, *J* = 5.2 Hz, 1H), 5.20 (d, *J* = 5.2 Hz, 1H), 5.00-4.93 (m, 2H), 4.45-4.15 (m, 1H), 4.01-3.91 (m, 1H), 3.84 (s, 3H), 3.61 (s, 3H), 3.19-3.14 (m, 1H), 3.09-2.84 (m, 4H), 2.77-2.64 (m, 2H), 2.59-2.53 (m, 1H).

^{13}C NMR (100 MHz, $CDCl_3$): δ 212.1, 151.0, 145.6, 133.7, 130.2, 130.1, 123.8, 118.5, 112.2, 99.9, 72.8, 58.0, 56.0, 54.5, 43.9, 39.5, 32.7, 29.0.

IR: ν (cm^{-1}) 2943 (w), 2158 (m), 1712 (m), 1552 (s), 1485 (m), 1448 (m), 1381 (m), 1346 (w), 1269 (m), 1165 (m), 1045 (m), 974 (m), 926 (m), 806 (w), 758 (s).

$[\alpha]_D^{24}$ 45.1 (c 0.65, $CHCl_3$).

HRMS: (ESI/QTOF) m/z: $[M + Na]^+$ Calcd for $C_{18}H_{23}NNaO_6^+$ 372.1418; Found 372.1423.

Synthesis of compound 38

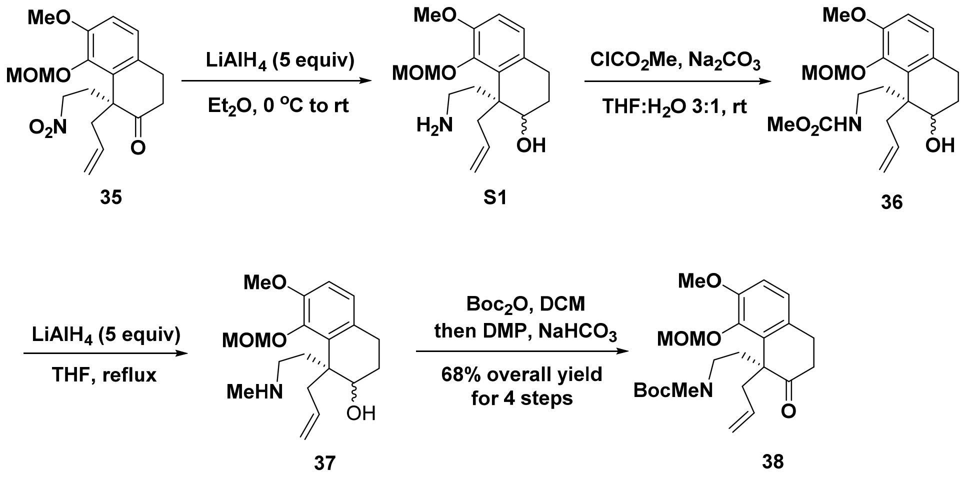

***tert*-butyl (*R*)-(2-(1-allyl-7-methoxy-8-(methoxymethoxy)-2-oxo-1,2,3,4-tetrahydronaphthalen-1-yl)ethyl)(methyl)carbamate:**[8] Under N_2 atmosphere, to a solution of compound **35** (800 mg, 2.3 mmol, 1.0 equiv) in dry Et_2O (100 mL) was added $LiAlH_4$ (437 mg, 11.5 mmol, 5.0 equiv) at 0 ºC, then the reaction mixture was warmed to room temperature slowly and stirred overnight. The reaction mixture was cooled to 0 ºC and quenched with sodium sulfate decahydrate (9.3 g, 28.8 mmol,). After stirring for 0.5 h at room temperature, the reaction mixture was filtered, washed with DCM and concentrated *in vacuo* to give the crude product **S1**, which was used directly without further purification.

To a solution of the crude **S1** in mixed solvent THF/H_2O (40 mL, $THF:H_2O$ = 3:1) was added Na_2CO_3 (732 mg, 6.9 mmol, 3.0 equiv) and methyl chloroformate (0.36 mL, 4.6 mmol, 2.0 equiv) and the reaction mixture was stirred at room temperature for 3 h. The reaction mixture was extracted with ethyl acetate. The combined organic extracts were washed with brine, dried over Na_2SO_4, filtered and concentrated *in vacuo* to give the crude product **36**, which was used directly without further purification.[9]

Under N_2 atmosphere, to a solution of the crude compound **36** in dry THF (100 mL) was added $LiAlH_4$ (437 mg, 11.5 mmol, 5.0 equiv) at 0 ºC, then the reaction mixture was heated to reflux and stirred for 2 h. The reaction mixture was cooled to 0 ºC and quenched with sodium sulfate decahydrate (9.3 g, 28.8 mmol,). After stirring for 0.5 h at room temperature, the reaction mixture was filtered, washed with DCM and concentrated *in vacuo* to give the crude product **37**, which was used directly without further purification.[10]

To a solution of the crude **37** in DCM (50 mL) was added Boc_2O (501 mg, 2.3 mmol, 1.0 equiv) and the reaction mixture was stirred at room temperature overnight. The reaction mixture was cooled to 0 ºC and added $NaHCO_3$ (966 mg, 11.5 mmol, 5.0 equiv) and DMP (975 mg, 2.3 mmol, 1.0 equiv) sequentially. The reaction mixture was warmed to room temperature and stirred for 2 h. The reaction

was quenched with saturated aqueous $Na_2S_2O_3$ and extracted with ethyl acetate. The combined organic extracts were washed with brine, dried over Na_2SO_4, filtered and concentrated *in vacuo*. The crude product was purified by flash column chromatography (SiO_2, PE/AcOEt 4:1) to yield the pure product **38** (602 mg, 68% for 4 steps) as a yellow oil.[11]

^{1}H NMR (400 MHz, CDCl$_3$): δ 6.79 (br s, 2H), 5.46-5.36 (m, 1H), 5.29-5.15 (m, 2H), 4.92-4.82 (m, 2H), 3.80 (s, 3H), 3.60 (s, 3H), 3.20 (dd, *J* = 13.6, 6.0 Hz, 1H), 3.0-2.76 (m, 3H), 2.71 (s, 3H), 2.74-2.60 (m, 2H), 2.60-2.50 (m, 2H), 2.50-2.32 (m, 2H), 1.35 (s, 9H).

^{13}C NMR (100 MHz, CDCl$_3$): δ 213.3, 155.6, 151.0, 145.5, 134.7, 131.6, 130.4, 123.3, 117.6, 111.4, 99.5, 79.2, 57.8, 55.9, 55.2, 45.8 (major rotamer), 45.1 (minor rotamer), 43.6, 40.1, 35.3 (major rotamer), 34.6 (minor rotamer), 33.7, 28.6, 28.4.

IR: ν (cm^{-1}) 2941 (w), 2156 (m), 2023 (w), 1691 (s), 1516 (w), 1481 (m), 1446 (m), 1394 (m), 1365 (m), 1265 (s), 1161 (s), 1078 (m), 1036 (m), 974 (s), 918 (m), 877 (m), 798 (m), 758 (m), 721 (m), 673 (m)

$[\alpha]_D^{23}$ -18.8 (c 0.77, $CHCl_3$).

HRMS: (ESI/QTOF) m/z: $[M + Na]^+$ Calcd for $C_{24}H_{35}NNaO_6^+$ 456.2357; Found 456.2356.

Synthesis of compound 39

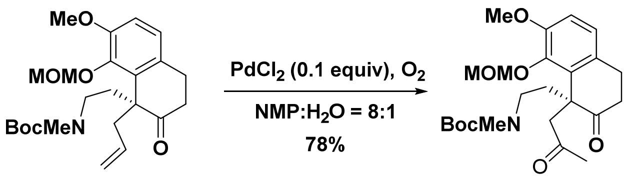

38 39

***tert*-butyl (*S*)-(2-(7-methoxy-8-(methoxymethoxy)-2-oxo-1-(2-oxopropyl)-1,2,3,4-tetrahydronaphthalen-1-yl)ethyl)(methyl)carbamate:[12]** Under O_2 atmosphere, to a solution of compound **38** (300 mg, 0.69 mmol, 1.0 equiv) in mixed solvent NMP/H_2O (18.0 mL, NMP:H_2O = 8:1) was added $PdCl_2$ (12.2 mg, 0.069 mmol, 0.1 equiv) and the reaction mixture was stirred at room temperature overnight. The reaction mixture was diluted with water and extracted with ethyl acetate. The combined organic extracts were washed with brine, dried over Na_2SO_4, filtered and concentrated *in vacuo*. The crude product was purified by flash column chromatography (SiO_2, PE/AcOEt 3:1) to yield the pure product **39** (242 mg, 78%) as a yellow oil.

^{1}H NMR (400 MHz, CDCl$_3$): δ 6.85 (d, *J* = 8.2 Hz, 1H), 6.76 (d, *J* = 8.3 Hz, 1H), 5.28-5.19 (m, 1H), 5.18-5.06 (m, 1H), 4.03 (d, *J* = 18.4 Hz, 1H), 3.79 (s, 3H), 3.61 (s, 3H), 3.47 (d, *J* = 18.6 Hz, 1H), 3.48-

3.33 (m, 1H), 3.15-2.90 (m, 2H), 2.90-2.77 (m, 2H), 2.72 (s, 3H), 2.68-2.52 (m, 1H), 2.40-2.22 (m, 1H), 2.22-2.02 (m, 1H), 1.98 (s, 3H), 1.38 (s, 9H).

^{13}C NMR (100 MHz, $CDCl_3$): δ 213.0, 206.9, 155.5, 150.6, 144.9, 131.9, 130.7, 123.7, 111.1, 99.8, 79.5, 57.9, 55.8, 51.9, 50.6, 45.7, 39.1, 36.5, 34.0, 29.5, 29.2, 28.4.

IR: ν (cm^{-1}) 2925 (m), 2359 (m), 1693 (s), 1483 (m), 1441 (m), 1396 (m), 1363 (m), 1269 (m), 1157 (s), 1045 (s), 978 (s), 924 (m), 881 (m)

$[\alpha]_D^{22}$ -41.8 (c 0.59, $CHCl_3$).

HRMS: (ESI/QTOF) m/z: $[M + Na]^+$ Calcd for $C_{24}H_{35}NNaO_7^+$ 472.2306; Found 472.2301.

Synthesis of compound 26

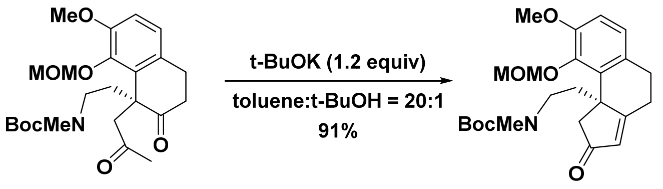

39 26

***tert*-butyl (*R*)-(2-(8-methoxy-9-(methoxymethoxy)-2-oxo-1,2,4,5-tetrahydro-9bH-cyclopenta[a]naphthalen-9b-yl)ethyl)(methyl)carbamate:**[13] Under N_2 atmosphere, to a solution of compound **39** (130 mg, 0.29 mmol, 1.0 equiv) in mixed solvent toluene/t-BuOH (10.5 mL, toluene: t-BuOH = 20:1) was added t-BuOK (42.5 mg, 0.35 mmol, 1.2 equiv) at 0 ºC and the mixture was warmed to room temperature slowly and stirred for 1 h. The reaction mixture was diluted with water and extracted with ethyl acetate. The combined organic extracts were washed with brine, dried over Na_2SO_4, filtered and concentrated *in vacuo*. The crude product was purified by flash column chromatography (SiO_2, PE/AcOEt 3:1 to 2:1) to yield the pure product **26** (114 mg, 91%) as a yellow oil.

^{1}H NMR (400 MHz, $CDCl_3$): (2 rotamers) δ 6.78 (br s, 2H), 6.02 (s, 1H), 5.24 (d, J = 5.0 Hz, 1H), 5.15 (d, J = 5.0 Hz, 1H), 3.80 (s, 3H), 3.61 (s, 3H), 3.35-2.55 (m, 6H), 2.99 (d, J = 18.9 Hz, 1H), 2.80 (d, J = 18.9 Hz, 1H), 2.78 (s, 3H), 2.56 (td, J = 12.9, 4.0 Hz, 1H), 2.04-1.82 (m, 1H), 1.41 (s, 9H).

^{13}C NMR (100 MHz, $CDCl_3$): rotamers δ 207.7, 182.9, 182.4, 155.5, 150.7, 144.3, 136.0, 127.9, 127.5, 127.3, 124.0, 111.6, 99.3, 79.6, 79.3, 57.8, 56.0, 49.7, 47.6, 45.1, 44.9, 36.4, 35.7, 34.4, 34.0, 31.1, 28.5, 25.7.

IR: ν (cm^{-1}) 2937 (w), 2359 (w), 1691 (s), 1631 (m), 1485 (m), 1396 (m), 1273 (m), 1246 (m), 1163 (s), 1078 (m), 1047 (m), 985 (w), 924 (m), 879 (w), 758 (m), 654 (m).

$[\alpha]_D^{23}$ -136.9 (c 0.86, $CHCl_3$).

HRMS: (ESI/QTOF) m/z: $[M + Na]^+$ Calcd for $C_{24}H_{33}NNaO_6^+$ 454.2200; Found 454.2204.

Synthesis of compound 40

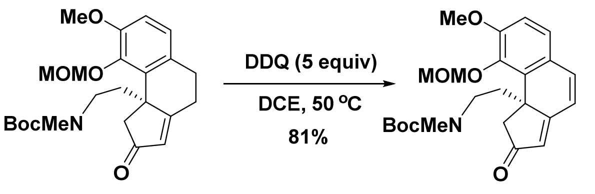

***tert*-butyl (*R*)-(2-(8-methoxy-9-(methoxymethoxy)-2-oxo-1,2-dihydro-9bH-cyclopenta[a]naphthalen-9b-yl)ethyl)(methyl)carbamate:**[14] To a solution of compound **26** (100 mg, 0.23 mmol, 1.0 equiv) in DCE (10.0 mL) was added DDQ (261 mg, 1.15 mmol, 5.0 equiv). The reaction mixture was heated to 50 °C and stirred at that temperature for 24 h. The reaction was quenched with 1 M NaOH (5 mL) and the mixture was extracted with ethyl acetate. The combined organic extracts were washed with brine, dried over Na_2SO_4, filtered and concentrated *in vacuo*. The crude product was purified by flash column chromatography (SiO_2, PE/AcOEt 2:1) to yield the pure product **40** (80 mg, 81%) as an amorphous yellow solid.

^{1}H NMR (400 MHz, $CDCl_3$): δ 6.96 (d, J = 8.0 Hz, 1H), 6.78 (d, J = 8.3 Hz, 1H), 6.69 (d, J = 9.5 Hz, 1H), 6.62 (d, J = 9.5 Hz, 1H), 5.90 (s, 1H), 5.20 (d, J = 4.0 Hz, 1H), 5.15 (d, J = 4.0 Hz, 1H), 3.85 (s, 3H), 3.61 (s, 3H), 3.07, 3.07 (ABq, J = 17.9 Hz, 2H), 3.04-2.94 (m, 2H), 2.67 (s, 3H), 2.18-1.87 (m, 2H), 1.34 (s, 9H).

^{13}C NMR (100 MHz, $CDCl_3$): (major rotamer) δ 207.9, 176.6, 155.4, 153.2, 144.4, 135.3 (2C), 126.2, 125.6, 124.9, 120.0, 110.6, 99.5, 79.3, 58.1, 55.9, 50.4, 48.2, 45.7, 42.4, 34.1, 28.4.

IR: ν (cm^{-1}) 2931 (w), 1685 (s), 1608 (m), 1574 (m), 1552 (w), 1479 (m), 1439 (m), 1394 (m), 1365 (m), 1265 (s), 1151 (s), 1080 (m), 1038 (m), 924 (s), 877 (m), 825 (m), 773 (m), 733 (m)

$[\alpha]_D^{21}$ -1023.2 (c 1.0, $CHCl_3$).

HRMS: (ESI/QTOF) m/z: $[M + Na]^+$ Calcd for $C_{24}H_{31}NNaO_6^+$ 452.2044; Found 454.2035.

Synthesis of compound 43

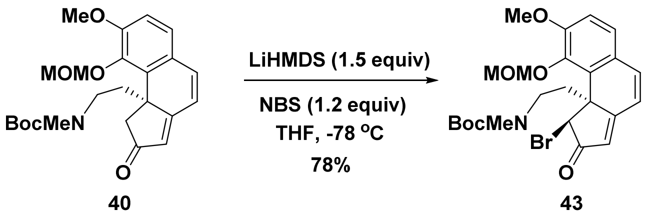

tert-butyl (2-((1*S*,9b*R*)-1-bromo-8-methoxy-9-(methoxymethoxy)-2-oxo-1,2-dihydro-9bH-cyclopenta[a]naphthalen-9b-yl)ethyl)(methyl)carbamate: Under N_2 atmosphere, to a solution of compound **40** (30 mg, 0.07 mmol, 1.0 equiv) in dry THF (3.0 mL) was added LiHMDS (0.105 mL, 0.105 mmol, 1.0 M in THF, 1.5 equiv) dropwise at -78 °C and the mixture was stirred at that temperature. After one hour, a solution of NBS (15.0 mg, 0.084 mmol, 1.2 equiv) in THF (1.0 mL) was added to the reaction mixture and the mixture was stirred for 1 h. The reaction was quenched with saturated $Na_2S_2O_3$ and extracted with ethyl acetate. The combined organic extracts were washed with brine, dried over Na_2SO_4, filtered and concentrated *in vacuo*. The crude product was purified by flash column chromatography (SiO_2, PE/AcOEt 2:1) to yield the pure product **43** (27.7 mg, 78%) as an amorphous yellow solid.

^{1}H NMR (400 MHz, CDCl$_3$): δ 7.04-6.94 (m, 1H), 6.85 (d, *J* = 8.4 Hz, 1H), 6.78-6.69 (m, 1H), 6.67 (d, *J* = 9.4 Hz, 1H), 5.91 (s, 1H), 5.46-5.20 (m, 2H), 4.93 (s, 1H), 3.87 (s, 3H), 3.65 (s, 3H), 3.30-2.98 (m, 1H), 2.80-2.54 (m, 2H), 2.63 (s, 3H), 1.64-1.52 (m, 1H), 1.35, 1.30 (two s, 9H).

^{13}C NMR (100 MHz, CDCl$_3$): (major rotamer) δ 202.0, 174.3, 155.3, 152.9, 144.5, 136.6, 131.3, 126.8, 125.5, 120.5, 120.2, 111.5, 99.6, 79.5, 58.0, 57.5, 55.9, 54.0, 45.8, 44.8, 34.1, 28.3.

IR: ν (cm^{-1}) 2978 (m), 2154 (m), 2079 (m), 1693 (s), 1603 (m), 1570 (m), 1479 (m), 1396 (m), 1269 (s), 1161 (s), 1011 (m), 910 (m), 864 (m), 818 (m), 785 (s), 760 (s), 731 (m), 717 (m), 665 (s).

$[\alpha]_D^{21}$ -1331.0 (c 1.0, CHCl$_3$).

HRMS: (ESI/QTOF) m/z: [M + Na]$^+$ Calcd for $C_{24}H_{30}BrNNaO_6^+$ 530.1149; Found 530.1155.

Synthesis of sinoracutine (9)

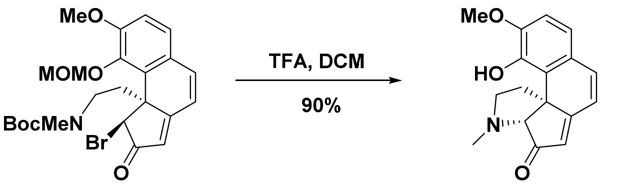

43

sinoracutine (9)

Sinoracutine: To a solution of compound **43** (25 mg, 0.05 mmol, 1.0 equiv) in DCM (3.0 mL) was added TFA (0.15 mL) at 0 ºC. The mixture was warmed to room temperature and stirred for 1 h. The reaction was quenched with saturated $NaHCO_3$ and the mixture was extracted with DCM. The combined organic extracts were washed with brine, dried over Na_2SO_4, filtered and concentrated *in vacuo*. The crude product was purified by flash column chromatography (SiO_2, DCM/MeOH/NEt_3 20:1:0.01) to yield the natural product sinoracutine (**9**) (12.7 mg, 90%) as an amorphous yellow solid.

One pot conversion of 40 to sinoracutine (9)

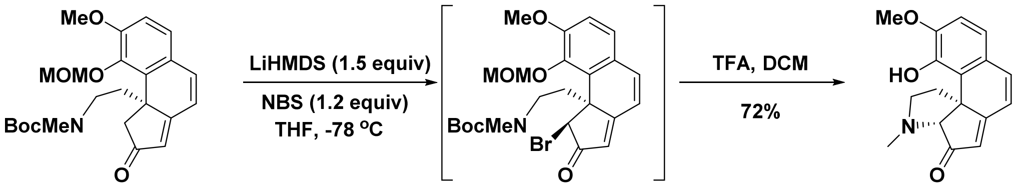

40 43 sinoracutine (9)

sinoracutine: Under N_2 atmosphere, to a solution of compound **40** (28 mg, 0.065 mmol, 1.0 equiv) in dry THF (3.0 mL) was added LiHMDS (0.98 mL, 0.98 mmol, 1.0 M in THF, 1.5 equiv) dropwise at -78 ºC and the mixture was stirred at that temperature. After one hour, a solution of NBS (13.9 mg, 0.078 mmol, 1.2 equiv) in THF (1.0 mL) was added to the reaction mixture and the mixture was stirred for 1 h. TFA (0.6 mL) and DCM (4 mL) were added. The reaction mixture was warmed to room temperature and stirred for 1 h. The reaction was quenched with saturated $NaHCO_3$ and the mixture was extracted with DCM. The combined organic extracts were washed with brine, dried over Na_2SO_4, filtered and concentrated *in vacuo*. The crude product was purified by flash column chromatography (SiO_2, DCM/MeOH/NEt_3 20:1:0.01) to yield the natural product sinoracutine (**9**) (13.2 mg, 72%) as an amorphous yellow solid.

^{1}H NMR (400 MHz, $CDCl_3$): δ 12.56 (br, 1H), 6.77 (d, J = 9.4 Hz, 1H), 6.75 (d, J = 8.3 Hz, 1H), 6.72 (d, J = 8.3 Hz, 1H), 6.54 (d, J = 9.4 Hz, 1H), 5.83 (s, 1H), 3.90 (s, 3H), 3.75 (s, 1H), 3.19-3.12 (m, 1H), 2.91 (s, 3H), 2.86-2.76 (m, 1H), 2.40-2.32 (m, 1H), 2.08 (ddd, J = 13.5, 10.7, 8.5 Hz, 1H).

^{13}C NMR (100 MHz, $CDCl_3$): δ 206.8, 175.0, 151.2, 145.7, 138.3, 127.7, 124.2, 123.4, 121.3, 118.1, 109.5, 72.4, 56.0, 53.8, 51.5, 41.7, 36.4.

IR: ν (cm^{-1}) 3591 (m), 3234 (m), 2951 (m), 2154 (s), 2023 (s), 1795 (m), 1768 (m), 1685 (m), 1516 (m), 1259 (s), 1223 (m), 756 (s), 737 (m), 719 (s), 683 (m), 663 (w).

$[\alpha]_D^{23.5}$ -1035.1 (c 0.77, $CHCl_3$) (94.4% ee) (lit: -1067.3 (c 0.35, $CHCl_3$) (99% ee)[15])

HRMS: (ESI/QTOF) m/z: $[M + H]^+$ Calcd for $C_{17}H_{18}NO_3^+$ 284.1281; Found 284.1277.

Synthesis of compound 44

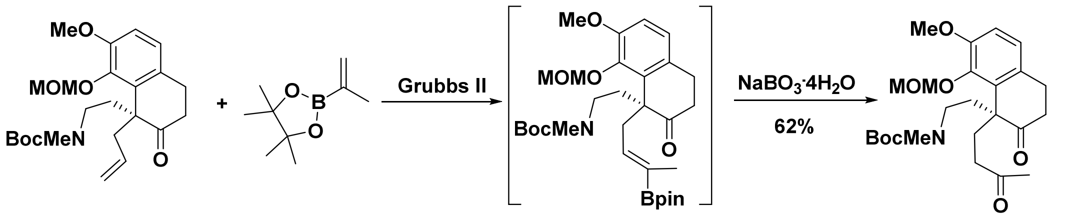

38 45 46 44

***tert*-butyl (*R*)-(2-(7-methoxy-8-(methoxymethoxy)-2-oxo-1-(3-oxobutyl)-1,2,3,4-tetrahydronaphthalen-1-yl)ethyl)(methyl)carbamate:[16]** Under Ar atmosphere, to a solution of compound **38** (200 mg, 0.46 mmol, 1.0 equiv) in dry DCM (2.3 mL) was added commercial available isopropenylboronic acid pinacol ester **45** (0.868 mL, 4.6 mmol, 10 equiv) and Grubbs 2nd generation catalyst (19.5 mg, 0.023 mmol, 0.05 equiv). The reaction mixture was heated to reflux and stirred at that temperature. After 24 h, additional Grubbs second generation catalyst (19.5 mg, 0.023 mmol, 0.05 equiv) was added and the mixture was stirred for 24 h. The reaction mixture was concentrated *in vacuo* and the residue was passed through a short column of silica gel to get the trisubstituted alkene product **46**.

To a solution of the above product in mixed slovent THF/H_2O (10 mL, THF:H_2O = 1:1) at ambient temperature was added sodium perborate tetrahydrate (354 mg, 2.3 mmol, 5.0 equiv) and the mixture was stirred for 30 min. The reaction mixture was extracted with ethyl acetate. The combined organic extracts were washed with brine, dried over Na_2SO_4, filtered and concentrated *in vacuo*. The crude product was purified by flash column chromatography (SiO_2, PE/AcOEt 2:1) to yield the pure product **44** (132 mg, 62%) as a colourless oil.

^{1}H NMR (400 MHz, $CDCl_3$): δ 6.86-6.78 (m, 2H), 5.30-5.10 (m, 2H), 3.81 (s, 3H), 3.59 (s, 3H), 3.00-2.68 (m, 4H), 2.73 (s, 3H), 2.62 (t, *J* = 7.1 Hz, 2H), 2.55 (ddd, *J* = 13.7, 11.3, 5.1 Hz, 1H), 2.46-2.35 (m, 3H), 2.13 (ddd, *J* = 16.7, 11.2, 5.1 Hz, 1H), 2.06-1.97 (m, 1H), 2.01 (s, 3H), 1.36 (s, 9H).

^{13}C NMR (100 MHz, CDCl$_3$): (major rotamer) δ 213.0, 208.2, 155.6, 151.1, 145.6, 131.9, 130.1, 123.5, 111.6, 99.6, 79.3, 57.9, 55.9, 54.1, 45.8, 40.0, 39.5, 35.7, 33.8, 31.6, 29.8, 29.0, 28.4.

IR: ν (cm^{-1}) 2925 (m), 2154 (s), 1695 (s), 1485 (s), 1437 (s), 1394 (s), 1365 (s), 1267 (s), 1167 (s), 1034 (s), 974 (s), 937 (s), 879 (s), 835 (s), 760 (s), 702 (s), 675 (s)

$[\alpha]_D^{22}$ +20.9 (c 0.82, CHCl$_3$).

HRMS: (ESI/QTOF) m/z: $[M + Na]^+$ Calcd for $C_{25}H_{37}NNaO_7^+$ 486.2462; Found 486.2464.

Synthesis of compound 27

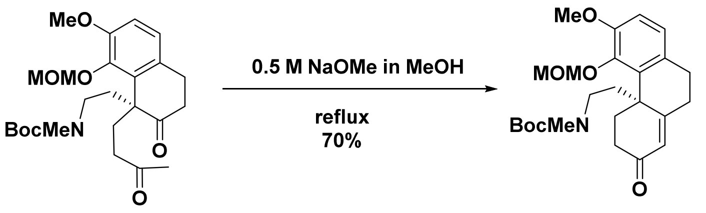

***tert*-butyl (*S*)-(2-(6-methoxy-5-(methoxymethoxy)-2-oxo-3,4,9,10-tetrahydrophenanthren-4a(2H)-yl)ethyl)(methyl)carbamate:[17]** Under N_2 atmosphere, a solution of compound **44** (97 mg, 0.21 mmol, 1.0 equiv) in dry MeOH (5.0 mL) was heated to reflux and a solution of NaOMe (0.5 mL, 2.7 mmol, 5.4 M in MeOH) was added dropwise. The reaction mixture was stirred at that temperature for 3 h and quenched with saturated NH_4Cl. The mixture was extracted with ethyl acetate. The combined organic extracts were washed with brine, dried over Na_2SO_4, filtered and concentrated *in vacuo*. The crude product was purified by flash column chromatography (SiO_2, PE/AcOEt 2:1 to 1:1) to yield the pure product **27** (65.4 mg, 70%) as a yellow oil.

^{1}H NMR (400 MHz, CDCl$_3$): δ 6.79 (br s, 2H), 5.89 (s, 1H), 5.30 (d, *J* = 4.2 Hz, 1H), 5.1 (br s, 1H), 3.81 (s, 3H), 3.63 (s, 3H), 3.37 (td, *J* = 12.5, 4.4 Hz, 1H), 3.16-3.09 (m, 1H), 3.00-2.91 (m, 1H), 2.90-2.68 (m, 4H), 2.78 (s, 3H), 2.68-2.52 (m, 2H), 2.52-2.40 (m, 1H), 2.28-2.08 (m, 1H), 2.00 (td, *J* = 13.8, 5.1 Hz, 1H), 1.38 (s, 9H).

^{13}C NMR (100 MHz, CDCl$_3$): (major rotamer) δ 199.5, 170.9, 155.6, 151.0, 145.6, 133.2, 129.8, 124.4, 124.0, 111.4, 99.6, 79.5, 57.8, 56.0, 47.3, 42.6, 37.3, 35.6, 34.9, 34.4, 34.0, 31.4, 28.5.

IR: ν (cm^{-1}) 2929 (m), 2339 (m), 1693 (s), 1481 (s), 1394 (s), 1163 (s), 1041 (s), 970 (s), 760 (s), 719 (s), 665 (s).

$[\alpha]_D^{20}$ -235.0 (c 0.70, CHCl$_3$).

HRMS: (ESI/QTOF) m/z: $[M + Na]^+$ Calcd for $C_{25}H_{35}NNaO_6^+$ 468.2357; Found 468.2367.

Synthesis of compound 47

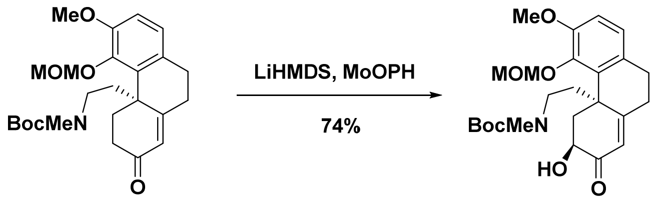

tert-butyl (2-((3*S*,4a*R*)-3-hydroxy-6-methoxy-5-(methoxymethoxy)-2-oxo-3,4,9,10-tetrahydrophenanthren-4a(2H)-yl)ethyl)(methyl)carbamate:[18] Under N_2 atmosphere, to a solution of compound **27** (30 mg, 0.067 mmol, 1.0 equiv) in dry THF (5.0 mL) was added LiHMDS (134 μL, 0.134 mmol, 2.0 equiv) at -78 °C. After 1 h, MoOPH (65 mg, 0.2 mmol, 3.0 equiv) was added. The reaction mixure was warmed to -20 °C slowly and stirred at that temperature for 5 h. The reaction mixture was quenched with saturated Na_2SO_3 and extracted with ethyl acetate. The combined organic extracts were washed with brine, dried over Na_2SO_4, filtered and concentrated *in vacuo*. The crude product was purified by flash column chromatography (SiO_2, PE/AcOEt 2:1 to 1:1) to yield the pure product **47** (22.8 mg, 74%) as a yellow oil.

^{1}H NMR (400 MHz, $CDCl_3$): δ 6.78 (br s, 2H), 5.98 (s, 1H), 5.27 (d, J = 5.0 Hz, 1H), 5.23 (d, J = 5.0 Hz, 1H), 4.63-4.54 (m, 1H), 3.80 (s, 3H), 3.66 (s, 3H), 3.57 (dd, J = 12.8, 5.6 Hz, 1H), 3.46 (br, 1H), 3.50-3.30 (m, 1H), 3.02-2.91 (m, 1H), 2.90-2.54 (m, 5H), 2.79 (s, 3H), 2.43-2.28 (m, 1H), 1.92 (t, J = 13.0 Hz, 1H), 1.40 (s, 9H).

^{13}C NMR (100 MHz, $CDCl_3$): (major rotamer, minor rotamer in parenthesis) δ 200.1, 172.4, 155.5, 151.0, 145.2, (133.6) 133.2, 129.1 (128.9), 124.0, 121.7, 111.7, 99.5, 79.6, 70.2, 58.0, 56.1, 47.3 (46.7), 44.6, 44.4, 37.9 (37.1), 34.1, 33.8 (33.6), 31.6, 28.5.

IR: ν (cm^{-1}) 2927 (m), 2156 (m), 1799 (s), 1682 (s), 1483 (s), 1394 (s), 1365 (s), 1271 (s), 1242 (s), 1163 (s), 1055 (s), 1041 (s), 1024 (s), 966 (s), 939 (s), 912 (s), 879 (s), 866 (s), 804 (s), 717 (s), 690 (s), 671 (s), 656 (s).

$[\alpha]_D^{21}$ -223.4 (c 0.70, $CHCl_3$).

HRMS: (ESI/QTOF) m/z: $[M + Na]^+$ Calcd for $C_{25}H_{35}NNaO_7^+$ 484.2306; Found 484.2310.

Synthesis of compound 48

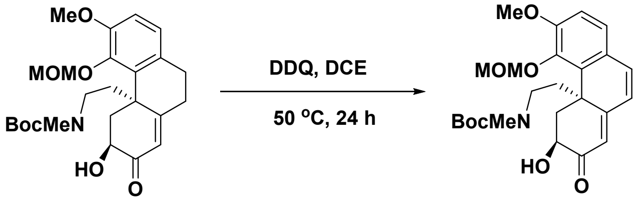
47 48

***tert*-butyl (2-((3*S*,4a*R*)-3-hydroxy-6-methoxy-5-(methoxymethoxy)-2-oxo-3,4-dihydrophenanthren-4a(2H)-yl)ethyl)(methyl)carbamate:**[14] To a solution of compound **47** (20 mg, 0.043 mmol, 1.0 equiv) in dry DCE (3.0 mL) was added DDQ (98.5 mg, 0.43 mmol, 10.0 equiv). The reaction mixture was heated to 50 °C and stirred at that temperature for 24 h. The reaction mixture was quenched with 1 M NaOH and extracted with ethyl acetate. The combined organic extracts were washed with brine, dried over Na_2SO_4, filtered and concentrated *in vacuo*. The crude product was purified by flash column chromatography (SiO_2, PE/AcOEt 1:1) to yield the pure product **48** (13.4 mg, 68%) as a yellow oil.

^{1}H NMR (400 MHz, $CDCl_3$): δ 6.96 (d, *J* = 7.8 Hz, 1H), 6.85 (d, *J* = 8.3 Hz, 1H), 6.70 (d, *J* = 8.9 Hz, 1H), 6.39 (d, *J* = 9.1 Hz, 1H), 5.95 (s, 1H), 5.32-5.10 (m, 2H), 4.72-4.58 (m, 1H), 4.09 (dd, *J* = 12.8, 5.4 Hz, 1H), 3.87 (s, 3H), 3.67 (s, 3H), 3.51 (s, 1H), 3.25-3.05 (m, 1H), 2.78-2.58 (m, 2H), 2.66 (s, 3H), 2.16-2.04 (m, 1H), 2.04-1.88 (m, 1H), 1.33 (s, 9H).

^{13}C NMR (100 MHz, $CDCl_3$): (major rotamer) δ 199.0, 165.7, 155.5, 153.7, 145.7, 135.4, 132.8, 126.4, 125.4, 125.2, 122.1, 111.1, 99.8, 79.4, 70.0, 58.2, 56.0, 45.5, 44.4, 42.8, 40.0, 34.1, 28.5.

IR: ν (cm^{-1}) 2979 (m), 2155 (s), 1796 (m), 1684 (m), 1558 (m), 1447 (m), 1396 (m), 1243 (m), 1154 (m), 1077 (m), 877 (m), 822 (m), 790 (m), 743 (m), 719 (s), 708 (m)

$[\alpha]_D^{21}$ -993.2 (c 0.33, $CHCl_3$).

HRMS: (ESI/QTOF) m/z: $[M + Na]^+$ Calcd for $C_{25}H_{33}NNaO_7^+$ 482.2149; Found 482.2154.

Synthesis of cepharatine A (5)

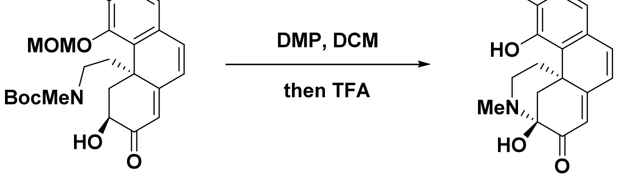
48 cepharatine A (5)

Cepharatine A: To a solution of compound **48** (25.6 mg, 0.056 mmol, 1.0 equiv) in DCM (3.0 mL) was added DMP (30.7 mg, 0.073 mmol, 1.3 equiv) at 0 °C. The reaction mixture was warmed to room temperature and stirred for 2 h. $Na_2S_2O_3$ (76 mg, 0.56 mmol) and TFA (0.5 mL) were added to the above reaction mixture. After stirring for another 1 h, DCM and TFA were removed *in vacuo* .The residue was diluted with saturated $NaHCO_3$ and extracted with ethyl acetate. The combined organic extracts were washed with brine, dried over Na_2SO_4, filtered and concentrated *in vacuo*. The crude product was purified by flash column chromatography (SiO_2, DCM/MeOH/NEt_3 100:5:1) to yield the pure product cepharatine A (**5**) (13.6 mg, 78%) as an orange foam.

^{1}H NMR (400 MHz, $CDCl_3$): δ 6.78 (d, J = 8.2 Hz, 1H), 6.76 (d, J = 8.2 Hz, 1H), 6.70 (d, J = 9.4 Hz, 1H), 6.29 (d, J = 9.4 Hz, 1H), 6.28 (br s, 1H), 6.12 (s, 1H), 4.31 (br s, 1H), 3.93 (s, 3H), 3.92 (d, J = 13.9 Hz, 1H), 2.91-2.84 (m, 1H), 2.74-2.58 (m, 2H), 2.24 (s, 3H), 2.22 (dd, J = 11.7, 2.7 Hz, 1H), 1.41-1.34 (m, 1H).

^{13}C NMR (100 MHz, $CDCl_3$): δ 194.2, 161.6, 148.2, 144.5, 136.1, 125.8, 125.6, 124.3, 123.6, 121.4, 108.8, 83.4, 56.4, 46.8, 44.5, 43.6, 36.4, 31.4.

IR: ν (cm^{-1}) 3234 (m), 2925 (m), 2274 (m), 2156 (m), 1944 (m), 1795 (m), 1649 (m), 1614 (m), 1558 (m), 1541 (m), 1520 (m), 1458 (m), 1373 (m), 1265 (m), 1076 (m), 1011 (m), 758 (s), 721 (m)

$[\alpha]_D^{21}$ -516.8 (c 0.29, $CHCl_3$) (lit: -537 (c 0.38, $CHCl_3$);[16] -716 (c 0.98, $CHCl_3$);[17])

HRMS: (ESI/QTOF) m/z: $[M + H]^+$ Calcd for $C_{18}H_{20}NO_4^+$ 314.1387; Found 314.1386.

Synthesis of cepharatine C (6)

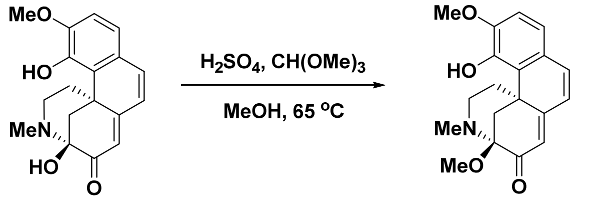

cepharatine A (5) cepharatine C (6)

Cepharatine C:[19] To a solution of cepharatine A (9.8 mg, 0.03 mmol) in MeOH (1.0 mL) were added trimethyl orthoformate (0.1 mL) and H_2SO_4 (0.1 mL of a 1 M solution in methanol). The reaction mixture was heated to 65 °C and stirred for 1 h at that temperature. After cooling to room temperature, the mixture was quenched with saturated $NaHCO_3$ and extracted with ethyl acetate. The combined organic extracts were washed with brine, dried over Na_2SO_4, filtered and concentrated *in vacuo*. The crude product was purified by flash column chromatography (SiO_2, DCM/MeOH/NEt_3 100:5:1) to yield the pure product cepharatine C (**6**) (9.3 mg, 95%) as an orange foam.

^{1}H NMR (400 MHz, $CDCl_3$): δ 6.79 (d, *J* = 8.2 Hz, 1H), 6.76 (d, *J* = 8.2 Hz, 1H), 6.65 (d, *J* = 9.3 Hz, 1H), 6.26 (d, *J* = 9.2 Hz, 1H), 6.26 (br s, 1H), 6.07 (s, 1H), 3.94 (d, *J* = 15.5 Hz, 1H), 3.94 (s, 3H), 3.38 (s, 3H), 2.90-2.80 (m, 1H), 2.67-2.54 (m, 2H), 2.20 (s, 3H), 2.15 (dd, *J* = 12.4, 2.6 Hz, 1H), 1.41-1.36 (m, 1H).

^{13}C NMR (100 MHz, $CDCl_3$): δ 191.8, 158.2, 147.9, 144.4, 135.1, 126.9, 126.1, 125.7, 123.8, 121.3, 108.9, 87.6, 56.4, 48.8, 46.7, 43.8, 39.0, 36.7, 31.5.

^{1}H NMR (400 MHz, CD_3OD): δ 6.90 (d, *J* = 8.2 Hz, 1H), 6.80 (d, *J* = 7.8 Hz, 1H), 6.78 (d, *J* = 8.9 Hz, 1H), 6.32 (d, *J* = 9.4 Hz, 1H), 6.04 (s, 1H), 4.16 (d, *J* = 12.4 Hz, 1H), 3.91 (s, 3H), 3.33 (s, 3H), 2.88-2.83 (m, 1H), 2.68 (td, *J* = 13.0, 5.2 Hz, 1H), 2.53 (td, *J* = 12.2, 3.4 Hz, 1H), 2.12 (s, 3H), 2.04 (dd, *J* = 12.4, 2.4 Hz, 1H), 1.35-1.32 (m, 1H).

^{13}C NMR (100 MHz, CD_3OD): δ 193.6, 162.6, 150.8, 146.5, 137.8, 126.81, 126.78, 126.5, 123.7, 122.6, 110.5, 88.9, 56.6, 47.8, 45.4, 40.2, 36.9, 32.1.

IR: ν (cm^{-1}) 2935 (m), 2156 (m), 2023 (m), 1876 (m), 1770 (m), 1658 (s), 1610 (s), 1566 (s), 1483 (s), 1442 (s), 1275 (s), 1242 (s), 1198 (s), 1086 (s), 1030 (s), 960 (s), 879 (s), 822 (s), 717 (s)

$[\alpha]_D^{23}$ -526.8 (c 0.21, MeOH) (lit: -550 (c 0.56, MeOH);[19] -332 (c 1.01, MeOH);[2])

HRMS: (nanochip-ESI/LTQ-Orbitrap) m/z: $[M + H]^+$ Calcd for $C_{19}H_{22}NO_4^+$ 328.1543; Found 328.1542.

Synthesis of compound 50

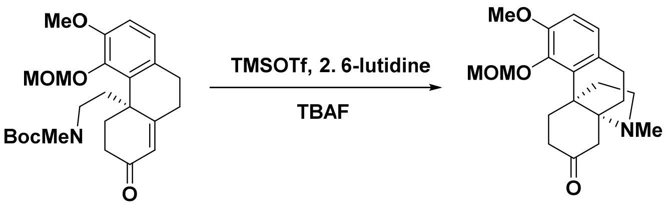

27 50

(4b*R*,8a*R*)-3-methoxy-4-(methoxymethoxy)-11-methyl-5,6,9,10-tetrahydro-8a,4b-(epiminoethano)phenanthren-7(8*H*)-one:[20] Under N_2 atmosphere, to a solution of **27** (110 mg, 0.25 mmol, 1.0 equiv) in DCM (10.0 mL) were added 2.6-lutidine (134 mg, 0.145 mL, 1.25 mmol, 5.0 equiv) and TMSOTf (0.136 mL, 0.75 mmol, 3.0 equiv) sequentially at 0 ºC. The reaction mixture was warmed to room temperature and stirred for 1 h. The reaction mixture was cooled to 0 ºC and TBAF (0.75 mL, 0.75 mmol, 1.0 M in THF, 3.0 equiv) was added. The mixture was stirred at that temperature for 20 minutes, then quenched with saturated $NaHCO_3$ and extracted with ethyl acetate. The combined organic extracts were washed with brine, dried over Na_2SO_4, filtered and concentrated *in vacuo*. The crude

product was purified by flash column chromatography (SiO_2, DCM/MeOH 200:1 to 100:1) to yield the pure product **50** (66 mg, 77%) as a yellow oil.

^{1}H NMR (400 MHz, $CDCl_3$): δ 6.78 (d, *J* = 8.4 Hz, 1H), 6.73 (d, *J* = 8.4 Hz, 1H), 5.22 (d, *J* = 4.8 Hz, 1H), 5.16 (d, *J* = 4.8 Hz, 1H), 3.81 (s, 3H), 3.63 (s, 3H), 2.90-2.68 (m, 3H), 2.68-2.52 (m, 4H), 2.49-2.32 (m, 3H), 2.22 (s, 3H), 2.11-1.94 (m, 2H), 1.68-1.56 (m, 2H).

^{13}C NMR (100 MHz, $CDCl_3$): δ 213.6, 150.8, 145.1, 138.4, 129.0, 123.7, 110.7, 99.1, 66.4, 57.7, 56.0, 51.1, 47.4, 46.2, 37.0, 36.3, 34.0, 33.4, 26.7, 24.8.

IR: ν (cm^{-1}) 2929 (m), 2852 (m), 2785 (w), 2158 (m), 2027 (w), 1716 (s), 1485 (s), 1444 (m), 1396 (m), 1273 (s), 1165 (s), 1078 (m), 1038 (m), 931 (s).

$[\alpha]_D^{22}$ -37.3 (c 0.4, $CHCl_3$).

HRMS: (ESI/QTOF) m/z: $[M + H]^+$ Calcd for $C_{20}H_{28}NO_4^+$ 346.2013; Found 346.2020.

Synthesis of compound 51

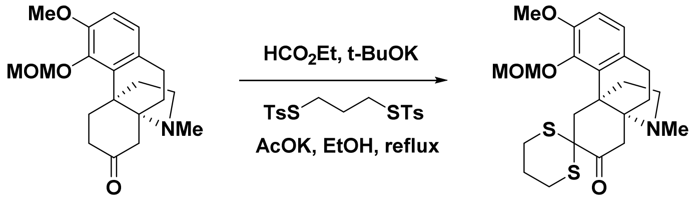

50 51

(4b'*S*,8a'*R*)-3'-methoxy-4'-(methoxymethoxy)-11'-methyl-9',10'-dihydro-5'*H*-spiro[[1,3]dithiane-2,6'-[8a,4b](epiminoethano)phenanthren]-7'(8'*H*)-one:[21] Under N_2 atmosphere, to a solution of compound **50** (56 mg, 0.16 mmol, 1.0 equiv) in Et_2O (6.0 mL) was added *t*-BuOK (36 mg, 0.32 mmol, 2.0 equiv) at 0 °C, then the reaction mixture was warmed to room temperature slowly and stirred for 2 h. Ethyl formate (0.6 mL) was added. After stirring for 2 h at room temperature, the reaction mixture was quenched with saturated NH_4Cl and extracted with ethyl acetate. The combined organic extracts were washed with brine, dried over Na_2SO_4, filtered and concentrated *in vacuo* to give the crude product, which was used directly without further purification.

To a solution of the above crude product in absolute MeOH (6.0 mL) was added trimethylene dithiotosylate (67 mg, 0.16 mmol, 1.0 equiv) and dry potassium acetate (157 mg, 1.6 mmol, 10.0 equiv) sequentially. The mixture was heated to reflux and stirred for 6 hours at that temperature. The reaction mixture was cooled to room temperature, quenched with water and extracted with ethyl acetate. The combined organic extracts were washed with brine, dried over Na_2SO_4, filtered and concentrated *in*

vacuo. The crude product was purified by flash column chromatography (SiO_2, $CHCl_3$/MeOH 100:1) to yield the pure product **51** (45 mg, 62%) as a brown oil.

^{1}H NMR (400 MHz, $CDCl_3$): δ 6.75 (br s, 2H), 5.32 (d, *J* = 5.0 Hz, 1H), 5.14 (d, *J* = 5.0 Hz, 1H), 3.79 (s, 3H), 3.68 (s, 3H), 3.27-3.16 (m, 2H), 3.04 (d, *J* = 15.2 Hz, 1H), 2.94 (d, *J* = 13.9 Hz, 1H), 2.89-2.77 (m, 2H), 2.79-2.60 (m, 5H), 2.54 (dt, *J* = 14.0, 3.4 Hz, 1H), 2.38 (dt, *J* = 13.9, 3.4 Hz, 1H), 2.26 (s, 3H), 2.28-2.18 (m, 1H), 2.06-1.99 (m, 1H), 1.78 (qt, *J* = 13.3, 3.0 Hz, 1H), 1.65 (t, *J* = 6.1 Hz, 2H).

^{13}C NMR (100 MHz, $CDCl_3$): δ 206.4, 150.7, 146.0, 136.7, 128.6, 123.9, 111.3, 99.4, 68.6, 58.0, 56.2, 52.6, 50.8, 47.5, 46.7, 41.8, 37.7, 33.4, 28.2, 26.3, 25.4, 24.6.

IR: ν (cm^{-1}) 3589 (s), 3078 (m), 2528 (m), 2364 (s), 2337 (m), 2156 (s), 2048 (m), 1795 (m), 1147 (m), 970 (m), 758 (s), 719 (s), 690 (m), 660 (s).

$[\alpha]_D^{22}$ +223.6 (c 0.3, $CHCl_3$).

HRMS: (ESI/QTOF) m/z: $[M + H]^+$ Calcd for $C_{23}H_{32}NO_4S_2^+$ 450.1767; Found 450.1771.

Synthesis of compound 52

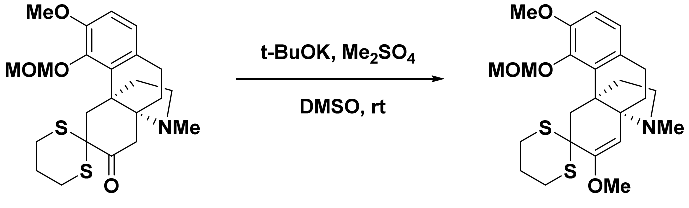

51 52

(4b'*S*,8a'*R*)-3',7'-dimethoxy-4'-(methoxymethoxy)-11'-methyl-9',10'-dihydro-5'*H*-spiro[[1,3]dithiane-2,6'-[8a,4b](epiminoethano)phenanthrene]:[22] Under N_2 atmosphere, to a solution of compound **51** (10 mg, 0.022 mmol, 1.0 equiv) in dry DMSO (0.5 mL) was added a solution of *t*-BuOK (7.5 mg, 0.067 mmol, 3.0 equiv) in DMSO (0.2 mL) at room temperature and the mixture was stirred at that temperature. After 5 minutes, a solution of Me_2SO_4 (3.0 mg, 0.0242 mmol, 1.1 equiv) in DMSO (0.1 mL) was added to the reaction mixture and the mixture was stirred for 1 h. The reaction was quenched with saturated NH_4Cl and extracted with ethyl acetate. The combined organic extracts were washed with brine, dried over Na_2SO_4, filtered and concentrated *in vacuo*. The crude product was purified by flash column chromatography (SiO_2, $CHCl_3$/MeOH 100:1 to 10:1) to yield the pure product **52** (8.6 mg, 85%) as an amorphous yellow solid.

^{1}H NMR (400 MHz, CD_3OD): δ 6.82 (d, *J* = 8.3 Hz, 1H), 6.72 (d, *J* = 8.4 Hz, 1H), 5.34 (d, *J* = 4.8 Hz, 1H), 5.12 (d, *J* = 4.8 Hz, 1H), 4.50 (s, 1H), 3.78 (s, 3H), 3.68 (s, 3H), 3.65 (s, 3H), 3.42 (ddd, *J* = 14.4, 11.4, 3.0 Hz, 1H), 3.17-3.08 (m, 1H), 3.11 (ddd, *J* = 13.9, 11.3, 2.7 Hz, 1H), 3.05-2.97 (m, 1H), 2.97-

2.88 (m, 1H), 2.80-2.65 (m, 5H), 2.49 (s, 3H), 2.51-2.43 (m, 1H), 2.18 (ddd, *J* = 14.0, 10.0, 5.7 Hz, 1H), 2.05-1.96 (m, 1H), 1.84-1.74 (m, 3H).

^{13}C NMR (100 MHz, CD_3OD): δ 166.9, 152.0, 147.0, 136.3, 130.5, 124.6, 112.9, 100.2, 92.5, 69.4, 58.1, 56.5, 55.3, 52.3, 47.2, 46.7, 45.7, 38.9, 34.8, 30.3, 29.3, 27.7, 27.3, 25.7.

IR: ν (cm^{-1}) 3234 (m), 2925 (m), 2852 (m), 2154 (s), 2022 (w), 1958 (w), 1821 (w), 1804 (w), 1795 (m), 1653 (m), 1449 (m), 1273 (m), 1164 (m), 1077 (m), 719 (s)

$[\alpha]_D^{22}$ -51.5 (c 0.3, $CHCl_3$).

HRMS: (ESI/QTOF) m/z: $[M+H]^+$ Calcd for $C_{24}H_{34}NO_4S_2^+$ 464.1924; Found 464.1935.

Synthesis of cepharamine (4)

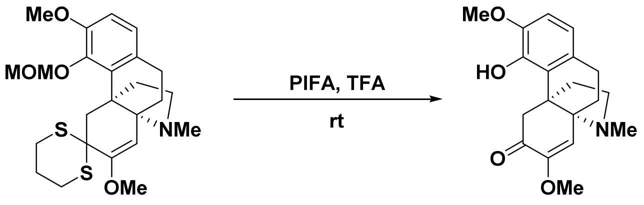

52 cepharamine (4)

Cepharamine:[23] To a solution of compound **52** (8.0 mg, 0.017 mmol, 1.0 equiv) in MeCN/H_2O (0.5/0.5 mL) was added TFA (0.0026 mL, 0.034 mmol, 2.0 equiv) and PIFA (18.3 mg, 0.0425 mmol, 2.5 equiv) at room temperature. After being stirred for 4 hours, $Na_2S_2O_3$ (5.4 mg, 0.034 mmol, 2.0 equiv) and TFA (0.05 mL) were added to the reaction mixture sequentially. The mixture was stirred for 1 h, quenched with saturated $NaHCO_3$ and extracted with ethyl acetate. The combined organic extracts were washed with brine, dried over Na_2SO_4, filtered and concentrated *in vacuo*. The crude product was purified by flash column chromatography (SiO_2, $CHCl_3$/MeOH 200:1 to 100:1) to yield the natural product cepharamine (**4**) (5.1 mg, 91%) as an amorphous yellow solid.

^{1}H NMR (800 MHz, $CDCl_3$): δ 6.69 (d, *J* = 8.2 Hz, 1H), 6.60 (d, *J* = 8.2 Hz, 1H), 5.96 (s, 1H), 5.62 (s, 1H), 3.85 (s, 3H), 3.73 (d, *J* = 16.7 Hz, 1H), 3.65 (s, 3H), 2.93-2.88 (m, 1H), 2.87 (td, *J* = 9.3, 3.3 Hz, 1H), 2.61-2.56 (m, 2H), 2.49 (d, *J* = 16.7 Hz, 1H), 2.41 (s, 3H), 2.35 (ddd, *J* = 10.0, 8.7, 6.8 Hz, 1H), 2.02-1.96 (m, 2H), 1.79 (td, *J* = 13.5, 4.8 Hz, 1H).

^{13}C NMR (200 MHz, $CDCl_3$): δ 194.3, 151.3, 145.2, 143.9, 129.0, 128.3, 119.6, 114.5, 108.8, 64.3, 56.4, 55.1, 51.7, 47.4, 44.5, 33.6, 33.5, 26.9, 25.1.

IR: ν (cm^{-1}) 3795 (w), 3730 (m), 3707 (m), 3606 (m), 3425 (m), 3128 (m), 2162 (s), 1641 (s), 1464 (s), 1267 (s), 1051 (s), 841 (s), 723 (s).

$[\alpha]_D^{26}$ -235.8 (c 0.3, $CHCl_3$).

HRMS: (ESI/QTOF) m/z: $[M+H]^+$ Calcd for $C_{19}H_{24}NO_4^+$ 330.1700; Found 330.1706.

Supplementary References:

[1] Bao, G.-H. et al. Sinoracutine, a novel skeletal alkaloid with cell-protective effects from Sinomenium acutum. *Tetrahedron Lett*. **50**, 4375-4377 (2009).

[2] He, L. et al. Cepharatines A−D, hasubanan-type alkaloids from *stephania cepharantha*. *J. Nat. Prod.* **74**, 181-184 (2011).

[3] Kashiwaba, N. et al. New Morphinane and Hasubanane Alkaloids from Stephania cepharantha. , *J. Nat. Prod*. **59**, 476-480 (1996).

[4] Chang, M-Y., Chan, C-K. & Lin, S-Y. One-pot access to 2-naphthols and benzofurans via the aerobic Wacker-type oxidation/intramolecular aldol cyclization. *Tetrahedron* **69**, 1532-1538 (2013).

[5] Tsang, K. & Brimble, M. Synthesis of aromatic spiroacetals related to γ-rubromycin based on a 3H-spiro[1-benzofuran-2,2′-chromane] skeleton. *Tetrahedron* **63**, 6015-6034 (2007).

[6] Rudolph, A. et al. Catalytic Asymmetric Conjugate Addition/Oxidative Dearomatization Towards Multifunctional Spirocyclic Compounds. *Angew. Chem. Int. Ed*. **50**, 5834-5838 (2011).

[7] Wang, S.-G. et al. Asymmetric dearomatization of β-naphthols through a bifunctional-thiourea-catalyzed michael reaction. *Angew. Chem. Int. Ed*. **54**, 14929-14932 (2015).

[8] Luo, S-P. et al. Toward the Total Synthesis of Haliclonin A: Construction of a Tricyclic Substructure. *Chem. Eur. J*. **19**, 87-91 (2013).

[9] Hicks, J. et al. Pd-Catalyzed N-Arylation of Secondary Acyclic Amides: Catalyst Development, Scope, and Computational Study. *J. Am. Chem. Soc*. **131**, 16720-16734 (2009).

[10] Snell, R., Woodward, R. & Willis, M. Catalytic Enantioselective Total Synthesis of Hodgkinsine B. *Angew. Chem. Int. Ed*. **50**, 9116-9119 (2011).

[11] Meyer, Y. et al. A comparative study of the self-immolation of para-aminobenzylalcohol and hemithioaminal-based linkers in the context of protease-sensitive fluorogenic probes. *Org. Biomol. Chem*. **8**, 1777-1780 (2010).

[12] Carcache, D. et al. "Total Synthesis of (±)-Jiadifenin and Studies Directed to Understanding Its SAR: Probing Mechanistic and Stereochemical Issues in Palladium-Mediated Allylation of Enolate-Like Structures. *J. Am. Chem. Soc*. **128**, 1016-1022 (2006).

[13] Kousara, M. et al. First enantioselective total synthesis and configurational assignments of suberosenone and suberosanone as potential antitumor agents. *Chem. Commun*. **51**, 3458-3461 (2015).

[14] Hartrampf, N. et al. Total synthesis of the norhasubanan alkaloid stephadiamine. *J. Am. Chem. Soc*. **140**, 8675-8680 (2018).

[15] Volpin, G., Vepřek, N. A., Bellan, A. B. & Trauner, D. Enantioselective synthesis and racemization of (−)-sinoracutine. *Angew. Chem. Int. Ed*. **56**, 897-901 (2017).

[16] Jung, M. E. & Yoo, D. First Total Synthesis of Rhodexin A. *Org. Lett*. **13**, 2698-2701 (2011).

[17] Broka, C. & Gerlits, J. Aziridinium cation mediated cyclizations. New routes to the morphinan ring system. *J. Org. Chem*. ***53***, 2144-2150 (1988).

[18] Zhu, L., Luo, J. & Hong, R. Total Synthesis of (±)-Cafestol: A Late-Stage Construction of the Furan Ring Inspired by a Biosynthesis Strategy. *Org. Lett*. **16**, 2162-2165 (2014).

[19] Chuang, K. V., Navarro, R. & Reisman, S. E. Short, enantioselective total syntheses of (−)-8-demethoxyrunanine and (−)-cepharatines A, C, and D. *Angew. Chem. Int. Ed*. **50**, 9447-9451 (2011).

[20] Sakaitani, M. & Ohfune, Y. Syntheses and reactions of silyl carbamates. 1. Chemoselective transformation of amino protecting groups via tert-butyldimethylsilyl carbamates. *J. Org. Chem*. **55**, 870-876 (1990).

[21] McMurry, J. & Farina, V. Total synthesis of O-methylpallidinine. *Tetrahedron Lett.* **24**, 4653-4656 (1983).

[22] Ahmad, N.et al. Synthesis of Polyprenylated Acylphloroglucinols Using Bridgehead Lithiation: The Total Synthesis of Racemic Clusianone and a Formal Synthesis of Racemic Garsubellin A. *J. Org. Chem*. **72**, 4803-4815 (2007).

[23] Stork, G. & Zhao, K. A simple method of dethioacetalization. *Tetrahedron Lett*. **30**, 287-290 (1989).
